# Modeling based insights into mechanical dysfunction in esophageal motility disorders

**DOI:** 10.1371/journal.pcbi.1013778

**Published:** 2025-12-26

**Authors:** Guy Elisha, Sourav Halder, Xinyi Liu, Dustin A. Carlson, Peter J. Kahrilas, John E. Pandolfino, Neelesh A. Patankar

**Affiliations:** 1 Department of Mechanical Engineering, Northwestern University, Evanston, Illinois, United States of America; 2 Division of Gastroenterology and Hepatology, Feinberg School of Medicine, Northwestern University, Chicago, Illinois, United States of America; 3 Kenneth C. Griffin Esophageal Center, Feinberg School of Medicine, Northwestern University, Chicago, Illinois, United States of America; 4 Department of Engineering Sciences and Applied Mathematics, Northwestern University, Evanston, Illinois, United States of America; Tecnológico de Monterrey: Tecnologico de Monterrey, MEXICO

## Abstract

Esophageal motility arises from the continuous coupling between enteric neural activity and the organ’s mechanical response, yet the structure of this coupling remains poorly understood. Esophageal motility disorders represent mechanical dysfunctions that originate from abnormalities in neural control, underscoring the need to understand how neural and mechanical processes interact to produce coordinated motion. We present an empirically guided neuromechanical model of the esophagus, comprising unidirectionally coupled relaxation oscillators activated by intrinsic enteric nervous system mechanoreceptors sensitive to wall distension. The model reveals complex behaviors emerging from interactions among its components, predicting various clinically observed normal and abnormal esophageal responses to distension. Specifically, repetitive antegrade contractions (RACs) are shown to arise from the coupled neuromechanical dynamics in response to sustained volumetric distension. Normal RACs are shown to have a robust balance between excitatory and inhibitory neural activities and mechanical input through these intrinsic distension-sensitive mechanoreceptors. When this balance is affected, contraction patterns resembling motility disorders emerge. For example, clinically observed repetitive retrograde contractions emerge due to hypersensitive mechanoreceptors in the esophageal wall. Such neuromechanical insights may ultimately guide the development of targeted pharmacological interventions.

## Author summary

The esophagus moves food and liquid to the stomach through waves of muscle contraction that are controlled by the neurons in esophageal walls. Problems in this process are known as esophageal motility disorders. The way neural signals and mechanical forces interact to generate these motions is still not well understood. In this work, we build a model that links how the esophageal wall stretches to how the local neurons respond to cause contractions. Using this model, we show how normal behavior such as repetitive antegrade (forward) contractions emerge naturally from the interaction of neural activity and mechanical distension. We also show how altering neural sensitivity or the balance between excitation and inhibition can produce contraction patterns that resemble clinically observed motility disorders, including repetitive retrograde (backward) contractions. By clarifying these mechanisms, our results may help guide future diagnostic approaches and targeted treatments.

## Introduction

An understanding of how neurological disorders lead to mechanical dysfunctions of organs remains an open problem [1,2]. Specifically, gastrointestinal and esophageal motility disorders (EMDs) are neurologically driven mechanical dysfunctions affecting approximately 35 million Americans [3–5]. These gaps in knowledge often lead to misinterpretations of disorders, impeding the development of effective neurologically focused treatment approaches [2,6]. Establishing a foundational understanding of the emergent behavior of organs may play a vital role in proposing targeted solutions.

Uncovering the pathogenesis of EMDs requires an understanding of the enteric nervous system (ENS) circuitry that controls peristalsis [7–9], a coordinated sequence of muscle contractions that propel content through the gastrointestinal tract [10,11]. Peristaltic motion is pervasive throughout the gastrointestinal tract, including the esophagus, stomach, and intestines [9,12,13]. In the esophagus, peristalsis can be triggered by deglutition (primary peristalsis), or locally, provoked by esophageal distension (secondary peristalsis), sensed by distension-sensitive mechanoreceptors [10,14–16].

The neuromuscular organization of the esophagus varies along its length: the proximal region consists of striated muscle, whereas the distal region consists of smooth muscle [17]. Peristalsis in the striated region depends on central mechanisms. Distension in the striated region activates vagal afferents, which elicit sequential discharge of vagal lower motor neurons in the nucleus ambiguus to drive contraction [10,18–20]. In the smooth muscle region, distension induced peristalsis is predominantly mediated by intrinsic enteric neural circuits [10]. Although vagal and spinal afferents provide modulatory input to this region, intrinsic circuitry is sufficient to generate and coordinate peristaltic activity in response to local distension [10,14,21]. Understanding how these neural circuits coordinate muscle activity is therefore essential for elucidating the mechanisms of peristalsis and its dysfunction. Despite its physiological importance, the precise organization of the ENS circuitry underlying secondary peristalsis in the esophagus has remained elusive [12,22,23].

Neuromechanical principles governing peristaltic transport have been extensively explored using clinical observations and computational modeling in other regions of the gastrointestinal tract, such as the small intestine, colon, and stomach [24–30]. In the colon, for example, neuromechanical loop models have been developed to describe the coupled dynamics between neural activation, wall deformation, and luminal flow [28–30]. These studies have shown that peristaltic coordination emerges from reciprocal feedback between mechanical distension and neural excitation, a concept that is likely conserved throughout the gastrointestinal tract, including the esophagus.

Within the gastrointestinal tract, including the esophagus, two primary types of motor neurons regulate muscle activity: excitatory neurons that induce contraction and inhibitory neurons that promote relaxation [31–33]. Although these general roles are established, the detailed structure and function of the esophageal enteric circuit remain poorly resolved [10]. Consequently, mechanistic models that isolate specific components of this circuitry are valuable for probing how local neural feedback and muscle mechanics interact to generate peristaltic motion.

Clinically, esophageal motility has been investigated with high-resolution manometry, a technology utilizing closely spaced pressure transducers positioned along the length of the esophagus [34]. High-resolution manometry is an excellent method for quantifying the strength and timing of esophageal contractions [35]. However, it cannot yield specific information about inhibition other than in tonically contracted sphincters [35,36].

**Fig 1:**
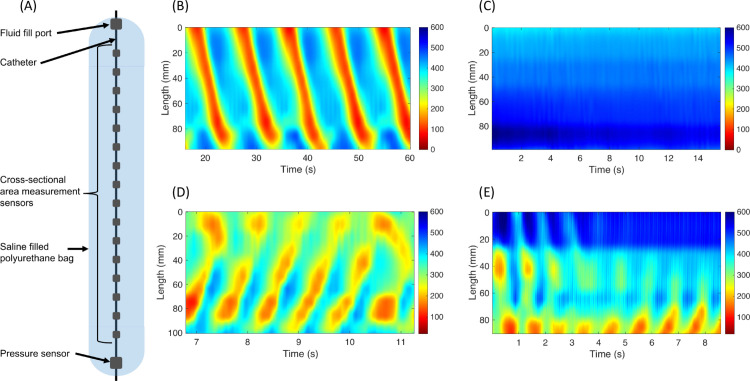
FLIP manometry and esophageal cross-sectional area topography. **(A)** Diagram of functional luminal imaging probe (FLIP) device. **(B, C, D,** and **E)** esophageal topography with color-coded cross-sectional area by axial length by time showing different distension-induced contractility patterns. **(B)** Repetitive antegrade contractions (RACs) pattern from an asymptomatic control. **(C)** Absent contractile response, **(D)** repetitive retrograde contractions, and **(E)** disordered contractions characterized by sporadic or chaotic pattern, not meeting antegrade nor retrograde contractions. Figures used with permission from the Esophageal Center at Northwestern University. The displayed topography represents data from the upper 60–70% of the FLIP recording, corresponding to the segment of the distal esophagus located above the lower esophageal sphincter. Although FLIP recordings include cross-sectional area measurements across the lower esophageal sphincter, these data were intentionally omitted since the focus of this study is on the esophageal body. The spatial extent of each map varies slightly because the position of the FLIP device relative to the sphincter differed across recordings, resulting in different y-axis limits. Note that FLIP records only from the smooth muscle portion of the esophagus; thus, the data shown do not include the proximal (striated muscle) region.

More recently, esophageal motility has been investigated using the functional lumen imaging probe (FLIP) manometry [37,38]. The FLIP device consists of a catheter surrounded by a fluid-filled bag that is positioned within the esophageal lumen to measure cross-sectional area at multiple locations over time (Fig 1A). In practice, FLIP recordings are typically performed in the distal (caudal) region of the human esophagus, which is composed of smooth muscle. The normal response elicited during a FLIP study (sustained volumetric distension) is of repetitive antegrade contractions (RACs, Fig 1B) [39,40]. FLIP studies exhibiting patterns other than RACs can also occur and are considered abnormal, potentially indicative of an EMD [37] (Figs 1C-E). Thus, FLIP has garnered attention as a potential diagnostic tool [38].

It has been proposed, based on clinical data, that the RACs pattern is a form of secondary peristaltic response to non-transient esophageal distension [39]. However, a complete understanding of how this pattern emerges and the involved mechanisms remains unresolved [41], and currently there is no known neural circuit that can explain this pattern [42]. Moreover, due to this lack of understanding, the emergence of abnormal patterns remains unclear [43–45]. Clarifying the connection between neural signals and observed mechanical dysfunctions can provide valuable insight into the nature of these disorders, crucial to developing targeted pharmacological interventions.

In this study, we propose a conceptually simple organ-scale neuromechanical model of the distal esophagus that is based on esophageal phenomenological studies, to predict and explain a broad repertoire of esophageal motility patterns. Organ-scale neuromechanical models have proven invaluable for unraveling the intricate patterns and behaviors that emerge from the interactions of individual components within a system, shedding light on how their failure leads to mechanical dysfunctions [27,46–50]. Through this model, our objective is to provide a theoretical framework capable of explaining the features observed in clinical FLIP studies. In addition, we use this model to reveal the underlying mechanisms associated with normal and abnormal FLIP contraction patterns, hopefully providing information on the pathogenesis of EMDs.

## Results

**Fig 2:**
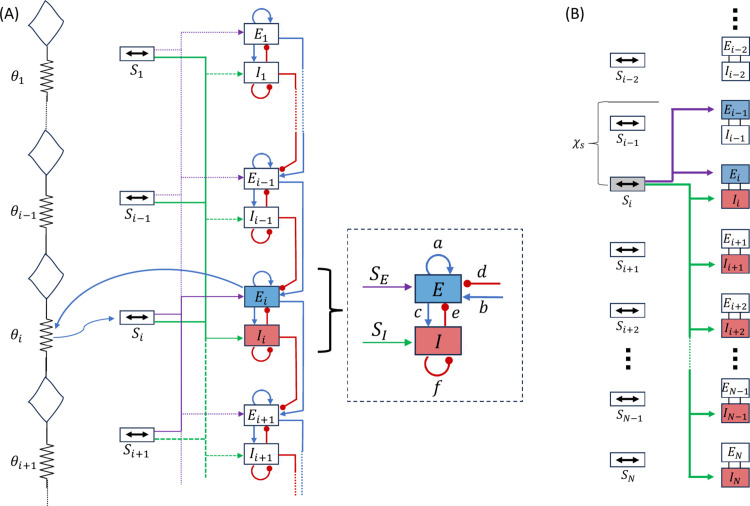
Schematic of the model. **(A)** An overview of the neuromechanical model consisting of the wall mechanics (chain of masses), neural circuitry (*E* and *I*), and the coupling dynamic (θ and *S*). The neural network is composed of *N* interconnected segments (Wilson-Cowan oscillators), each consisting of an excitatory (*E*) and an inhibitory (*I*) neuronal population. Lines with circular heads mark inhibitory synapses, and arrows denote excitatory synapses. The coupling mechanism is demonstrated on segment *i*, where distension activates mechanoreceptors (arrow from the muscle to the corresponding mechanoreceptors *S*_*i*_), and local excitation actuates local body segments (arrow from *E*_*i*_ to the corresponding muscle section). Parameters *a*,*b*,*c*,*d*,*e* and *f* represent the average synaptic weight of excitatory and inhibitory synapses per cell in the excitatory or inhibitory population. **(B)** Schematic showing the distension-induced excitation. When distension at *i* creates sufficient strain on the walls, the corresponding mechanoreceptor (*S*_*i*_) sends excitatory signal to caudal inhibitory populations and rostral excitatory populations. See S1 Text and Table A in S2 Text for additional details.

The results reported below are obtained based on the empirically guided neural circuit shown in Fig 2. The circuit and the corresponding neuromechanical mathematical model are described in the Methods section. The presentation of results is organized as follows: first, we reproduce regular RACs and explain their underlying mechanisms; second, we reproduce and interpret other clinically observed, distension-induced contractions; and finally, we demonstrate how the model captures and explains pathological conditions.

### Repetitive antegrade contractions

**Fig 3:**
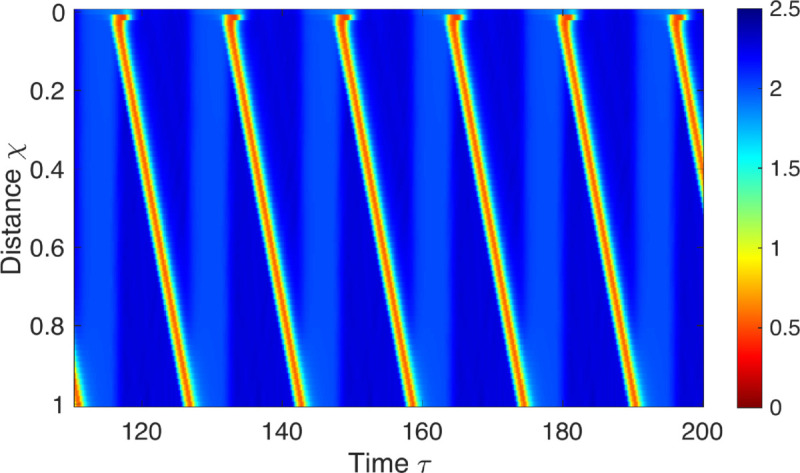
Esophageal spatio-temporal topography. Depicts non-dimensional color-coded cross-sectional area with respect to *A*_*o*_, obtained through a neuromechanical model simulation of FLIP manometry. χ is non-dimensional length with respect to *L* and τ is non-dimensional time with respect to $\tau_{E}$. The model exhibits a repetitive, antegrade contraction pattern autonomously triggered, mirroring observations from clinical FLIP measurements. The parametric values used to obtain these results are listed in Table 1.

Fig 3 presents a normal RACs pattern obtained through simulations of the mathematical model, qualitatively reproducing the key elements observed in RACs: repetitiveness, forward (antegrade) propagation, and non-overlapping contractions. The contractions emerge and are sustained autonomously and independently of any externally prescribed input. We establish Fig 3 as the baseline case. In this section, we use the model to explain the triggering mechanism of the pattern and the development of its essential elements, revealing the underlying dynamics of RACs.

#### Repetitiveness.

**Fig 4:**
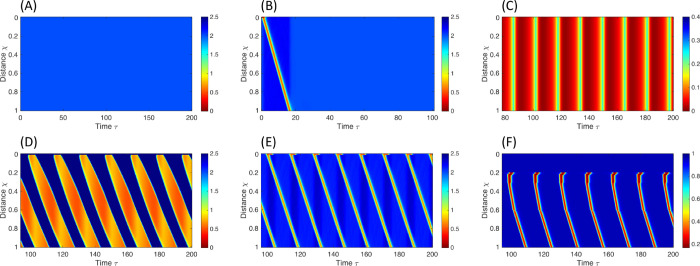
Color-coded spatiotemporal maps showing non-dimensional cross-sectional area (CSA), excitatory neuronal activity level, or muscle contraction pattern (θ), obtained from a neuromechanical model simulating esophageal distension tests. In the panels, χ and τ denote non-dimensional length and time, respectively, normalized by total esophageal length, *L*, and excitatory time constant, $\tau_{E}$. **(A)** CSA: Mechanoreceptors are disabled; the esophagus remains quiescent. Achieved by increasing the sensitivity threshold, $\hat{\alpha }$, or reducing sensory feedback to excitatory neurons, *w*_*E*_. **(B)** CSA: Mechanoreceptors are disabled, and a brief excitatory input (equivalent to *w*_*E*_) is applied at the rostral end, generating a single contraction before recovery. **(C)** Excitatory neuronal population activity: Eliminating neuronal neighboring connections by setting *b* = *d* = 0, resulting in uniform excitation along the esophagus. **(D)** CSA: Reducing inhibitory signaling from the anterior inhibitory population, *d*, increases excitation duration and produces overlapping contractions. **(E)** CSA: Lowering the excitatory activation threshold, $\phi_{E}$, generates early excitatory spikes and overlapping contractions. **(F)** Muscle contraction pattern θ: Simulated sustained esophageal distension using a short bag, but distension is set to activate inhibitory neurons locally only, such that *S*_*I*_ = 0 caudal to the bag. Parameter values for each simulation are listed in Tables A-E in S3 Text.

Excitatory signals from mechanoreceptors (*S*_*E*_) serve as the primary initiators of RACs and play a pivotal role in sustaining the repetitive pattern. Fig 4A demonstrates the consequence of disabling the mechanoreceptors, resulting in a lack of contractility due to insufficient excitatory input. Fig 4B illustrates a scenario in which the sensation of the mechanoreceptors is disabled, and a brief excitatory input is introduced at the rostral end of the smooth muscle section of the esophagus. In the model’s context, this is equivalent to setting $S_{E}=S_{I}=0$ everywhere except the rostral end, where $S_{E}\neq 0$ for a short time period. As depicted in Fig 4B, the rostral excitatory input travels down the esophageal length, leading to a single, propagating contraction. However, without the mechanoreceptors, there is no sustained excitatory input to initiate additional contractions.

The emergence of the repetitive, rhythmic attribute observed in FLIP studies is mathematically elucidated through the model. Consider section *i*, positioned along the length of the distended esophagus. Adequate sustained volumetric distension around *i* triggers the activation of local distension-sensitive mechanoreceptors. These receptors, in turn, excite excitatory neurons at *i* (via *S*_*E*,*i*_), initiating the excitatory phase. The activated excitatory neurons then stimulate both excitatory and inhibitory cells at location *i*. Over time, the activity of inhibitory cells surpasses a threshold level, overcoming both excitatory and inhibitory activity and inducing a refractory period where all cells cease activity. The sustained distension results in constant excitation, initiating a new cycle when all cells are inactive [51]. Note that distension-induced excitation also occurs in inhibitory pathways (*S*_*I*_), ensuring that inhibition precedes excitation.

#### Forward (antegrade) propagation.

The primary determinant governing the forward propagation of contractions is the presence of unidirectional connections (*b*), which create asymmetry in the total synaptic input received by the rostral oscillator [52,53]. In the absence of these connections, the system exhibits repetitive but non-propagating contractions (Fig 4C). The constant input from the mechanoreceptors to the excitatory population (*S*_*E*_) ensures that each oscillator operates at its natural frequency, generating an individual limit cycle. Therefore, rhythmic behavior persists even without unidirectional connections (Fig 4C).

Unidirectional coupling introduces an additional stimulus, which in an isolated case (considering a single oscillator in the chain) can be viewed as periodic perturbations. When a stable limit cycle experiences a perturbation, it introduces transient changes which quickly decay to the original oscillatory activity. However, it returns with a phase shift relative to its unperturbed cycle. This adjustment of the phase of each oscillator is termed phase resetting [54]. In a coupled system, the phase shift eventually stabilizes to a constant value for all subsequent perturbations, leading to a phase-locked state between coupled oscillators. The delay between each oscillator increases with distance from the first oscillator, as out-of-phase oscillations accumulate [52,55]. In other words, as each oscillator oscillates with a fixed phase lag relative to its proximal neighbor, the total phase difference between the first and subsequent oscillators grows linearly with position. Consequently, the signal propagates through the chain as a traveling wave, with each oscillator oscillating with a delay relative to its proximal neighbor [56,57]. Since muscle contraction follows excitatory signal’s pattern (Eq (8)), muscle contraction pattern appears as a propagating wave [32,58,59]. Note that the constant phase scenario is the solution to the system. The system is not each oscillator by itself; instead, it forms a new system - a coupled chain - with its own natural frequency and a phase difference between adjacent oscillators.

#### Non-overlapping.

Lastly, the absence of overlapping patterns observed in normal RACs results from a balance among various factors, including input to the excitatory population (such as *w*_*E*_ and *d*), inhibitory activity levels (controlled by *c*, *f*, and *w*_*I*_), and the excitatory activation threshold ($\phi_{E}$). A parametric study reveals that overlapping contractions occur when the firing of excitatory populations dominates inhibitory firing (see S4 Text). This leads to excitation in the rostral end before the refraction of the caudal end, observed in two distinct ways. The first involves extending the excitatory phase. This is exemplified, for instance, by reducing the inhibitory signal from the anterior inhibitory population (*d*) (Fig 4D). The second entails allowing excitatory activity to spike faster, effectively shortening the refractory period. This is demonstrated, for instance, by reducing the excitatory activation threshold ($\phi_{E}$), requiring less excitation to activate excitatory cells (Fig 4E).

### Comparison with clinical data

Numerous studies over the years explored esophageal responses to distension, offering insights into the typical reactions of the esophagus under various conditions. Similar to RACs, many of these responses are non-intuitive and their mechanisms have not been formally explained [14,16,60–63]. In this section, we showcase the capability of the proposed neural model to reproduce these diverse distension-induced esophageal scenarios, demonstrating its versatility beyond FLIP manometry. Importantly, we leverage the results to briefly explain these non-intuitive clinical observations in a healthy esophagus. We present four distinct scenarios representing a healthy esophageal response to distension:

**Case 1. Transient esophageal distension:** Results in a single contraction [62]. The short stimulus can be introduced through abrupt inflation and deflation of a distending medium or through vagal efferent nerve stimulation.
**Case 2. Prolonged esophageal distension using a short balloon followed by abrupt deflation:** During the distension, the esophagus caudal to the distended section is mostly inactive. Upon abrupt deflation, one phasic contraction appears, traveling down the length of the esophagus [62]. The pressure and cross-sectional area variations along the distended region are not reported in these studies.
**Case 3. Sustained distension of a short section of the esophagus:** Results in repetitive contractions along the distended section, as observed through a pressure probe located inside the fluid-filled distending bag [16].
  The contractions’ frequency remains independent of the bag’s length, exhibiting a consistent rate of 6 contractions per minute, akin to the FLIP scenario [16,41]. The pressure variations caudal to the distended region are not reported in these studies.
**Case 4. Sustained esophageal distension along its entire smooth muscle length:** Results in RACs [41,44].

When reproducing the above scenarios, the neural circuit and its parametric values remain constant across the different simulations. The objective is to demonstrate that the properties inherent in the baseline solution, representing a typical esophageal response to FLIP (Fig 3), automatically encompass all clinically observed distension-induced scenarios. The simulations vary by adjusting the distension’s location and duration along the esophagus.

**Fig 5:**
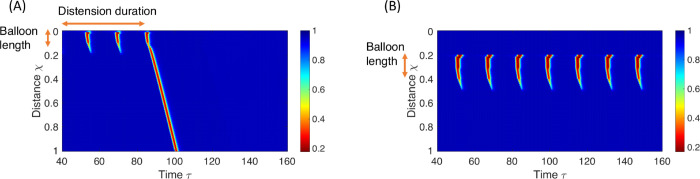
Color-coded spatio-temporal topographies depicting muscle contraction patterns (θ, non-dimensional by definition) obtained by the neuromechanical model, simulating two distinct esophageal distension tests. χ is non-dimensional length with respect to *L* and τ is non-dimensional time with respect to $\tau_{E}$. **(A)** Prolonged balloon distension followed by abrupt deflation (Case 2). **(B)** Sustained esophageal distension using a short bag (Case 3). The parametric values used for these simulations are displayed in Tables F and G in S4 Text.

All four scenarios are successfully reproduced. Cases 1 and 4 are displayed in Figs 4B and 3, respectively, and discussed in the preceding section. Fig 4B corresponds to Fig 1 in the manuscript by Paterson et al. [62]. Cases 2 and 3 are presented in Fig 5 and elaborated upon in the following discussion. Fig 5A corresponds to Fig 2 in the manuscript by Paterson et al. [62], and Fig 5B corresponds to Fig 1D in the manuscript by Gregersen et al. [16].

Note that cases 2 and 3, though similar, were reported by different research groups, conducting different studies. Paterson et al. [62] (Case 2) focused on the esophageal response caudal to the distension (air-filled balloon), providing detailed pressure readings solely caudal to the balloon. Gregersen et al. [16] (Case 3) documented the pressure and cross-sectional area only inside the distending medium. From these separate studies, we infer that sustained esophageal distension using a short bag or balloon induces repetitive contractions *only* along the distended section, with the rest of the esophagus mostly inactive. Upon abrupt removal of the distension, a single contraction emerges, traveling beyond the distended segment. Fig 5 displays the muscle contraction pattern over the length of the smooth muscle section of the esophagus, capturing the behavior inside and outside the bag/balloon.

To explain the clinical observations in cases 2 and 3, we specifically focus on unique aspects not discussed in the preceding section, including (i) the selective appearance of contractions solely along the distended section (with a quiescent state caudal to the bag), (ii) the observation of a traveling contraction upon deflation of the bag, and (iii) the explanation for the contraction frequency’s independence of the bag’s length.

(i) As previously established, repetitive muscle contractions at any location along the smooth muscle region of the esophagus are both initiated and sustained by local distension-sensitive mechanoreceptors that excite nearby excitatory neurons. Because this input is primarily local [31], regions not directly distended, such as those proximal or distal to the bag, receive little to no excitatory drive and thus remain quiescent (as illustrated in Fig 4A).
  While this explains the lack of contraction proximal to the distended segment, excitatory neurons can also receive input from other excitatory neurons located rostrally (parameter *b* in our model). This coupling allows excitation to propagate even in the absence of local distension, as shown in Fig 4B. Why, then, do these waves not continue propagating caudally beyond the distended region?
  The key factor is descending inhibition. When the esophagus is excited at any location, excitation is sent to inhibitory neurons caudally [64], causing the esophagus to relax in anticipation of an incoming bolus [10]. Thus, introducing enough inhibition stops the wave from propagating beyond the distended region. In our model, this is represented by $S_{I,i}\neq 0$, where *i* is a location caudal to the distended section. If we set the distension-induced excitation to inhibitory neurons only around the distended region ($\beta_{I}=\beta_{E}$), as in Fig 4F, contractions caudal to the distended region are present.
(ii) Note that the inhibitory activity levels caudal to distension are low, allowing a quick recovery once distension is eliminated. Conversely, excitatory activity levels along the distended section are high, taking longer to recover. Therefore, upon abrupt emptying of the bag or balloon, the excitatory signal is free to travel down through neighboring communication (parameter *b*), unimpeded by inhibitory activity (as depicted in Fig 4B). This propagating excitatory signal translates into muscle contraction, resulting in the observed phasic contraction.
(iii) Examining the rate of propagating contractions is equivalent to studying the frequency of muscle contraction at a single location, dependent on the excitation-refractory cycle of neuronal populations at that specific location [16,44]. The time elapsed from the initiation of the excitatory phase to the end of the refractory period is dictated by the neural architecture and the strength of excitatory input from the distension-sensitive mechanoreceptors (*w*_*E*_) [51]. Since these parameters are independent of the bag’s length, the contraction frequency is not expected to change with the bag’s length.

### Esophageal motility disorders

In this section, we showcase how alterations in specific parameters disrupt the RACs pattern. These disruptions result in solutions reminiscent of established esophageal motility disorders, as defined by the Chicago Classification [35,44]. This demonstration not only exposes the underlying mechanisms of these disorders but also emphasizes that they are emergent behaviors. Through this exploration, we establish the model’s consistency with pathologies, providing valuable insights into the dynamic interplay of components influencing esophageal motility disorders. The results are obtained through parametric and robustness studies available in S4 Text. Robust in this context refers to the system’s ability to maintain its behavior despite perturbations.

#### Absent contractile response.

As discussed earlier, an absent contractile response emerges when the excitatory population’s activity (*E*), responsible for controlling muscle contraction, is either reduced or absent (Fig 4A). This response may be attributed to dysfunctions in the normal operation of the distension-sensitive mechanoreceptors (*S*_*E*_ and *S*_*I*_), particularly when there are alterations in the sensitivity threshold of the mechanoreceptors ($\hat{\alpha }$). Changes in esophageal stiffness and dilation are suspected to impact esophageal sensitivity to distension, a phenomenon associated with motility disorders characterized by an absent contractile response, such as achalasia I [65].

The results in Fig 4B might provide insights into cases where patients exhibit normal primary peristalsis (as measured by high-resolution manometry) but abnormal findings in FLIP studies [36,39]. Such discrepancies could be explained by abnormal responses to distension, leading to a lack of an excitatory signal and, consequently, an inability to initiate secondary peristalsis.

#### Disordered non-occluding contractions.

**Fig 6:**
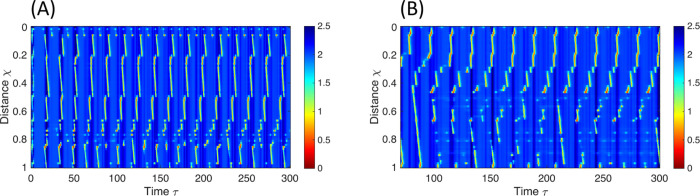
Spastic patterns obtained through a neuromechanical model mimicking esophageal FLIP test. The figures display the color-coded spatio-temporal topographies of non-dimensional cross-sectional area with respect to *A*_*o*_. χ is non-dimensional length with respect to *L* and τ is non-dimensional time with respect to $\tau_{E}$. **(A)** Chaotic response created by irregularities in inhibitory neuronal pathways (*e*). **(B)** Chaotic response to irregularities in excitatory input from the distension-sensitive mechanoreceptors (*w*_*E*_) with reduced inhibitory input to inhibitory pathways (see text). The parametric values used for these simulations are displayed in Tables H and I in S3 Text.

The introduction of local variations to the parameters defining the neuronal circuitry results in sporadic or chaotic contraction patterns, as illustrated in Fig 6. Given the inherent variability of biological systems, small irregularities are expected. Introducing minor parameter perturbations—implemented as Gaussian variations with small standard deviations around the baseline values—does not substantially alter the contraction pattern, demonstrating the model’s robustness (see Fig F in S4 Text). However, when the magnitude of local parameter variations increases or when the mean value of a key parameter (e.g., *e*) deviates from its baseline (e.g., reduced from 15 to 12), the excitatory–inhibitory balance required for proper RACs is disrupted, and the coordinated antegrade contractions break down, giving rise to pronounced irregularities and spastic patterns (Fig 6).

It is crucial to emphasize the significance of the system’s robustness. If the system is not initially robust, it becomes more sensitive to small irregularities. Such cases have parametric values that allow the solution to exhibit regular RACs. Nevertheless, the excitatory-inhibitory balance is not as stable. Thus, these scenarios are more prone to sporadic or chaotic contraction patterns when introducing small irregularities, which would not otherwise trigger such responses (Fig 6B).

#### Sustained panesophageal contractions.

**Fig 7:**
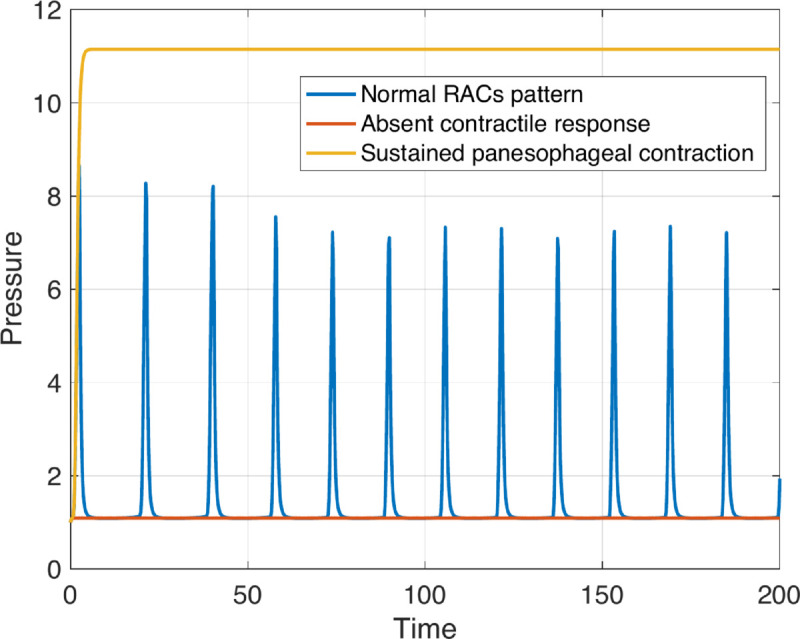
Internal bag pressure over time. Non-dimensional internal bag pressure (with respect to *K*_*e*_) over non-dimensional time (with respect to $\tau_{E}$) obtained in three distinct scenarios. Pressure is recorded at a single location along the bag length. In the case of normal RACs, the bag pressure fluctuates, reaching its peak during maximum contraction. Conversely, in absent contractile response, where no contraction occurs, the pressure remains consistently low throughout volumetric distension. In sustained panesophageal contraction, the entire esophagus contracts uniformly, increasing bag pressure that persists at a high level throughout the volumetric distension. The parametric values used to obtain these results are listed in Table 1 and Tables A and J in S3 Text.

Sustained, non-propagating contractions leading to an increase in FLIP pressure are observed when the inhibitory signal to the excitatory population is weakened. Thus, inhibitory activity is insufficient to overcome excitatory activity, allowing excitatory cells to remain active. This scenario is reproduced by reducing the inhibitory signal to adjacent excitatory neurons (*e*) or by decreasing the excitation of inhibitory neurons (*c* or *w*_*I*_). Note that since the FLIP’s fluid is incompressible, the color-coded topography of cross-sectional area may resemble absent contractile response (Fig 4A). However, the pressure profile differs, as the uniform muscle contraction increases the pressure in the bag, as illustrated in Fig 7.

#### Repetitive retrograde contractions.

**Fig 8:**
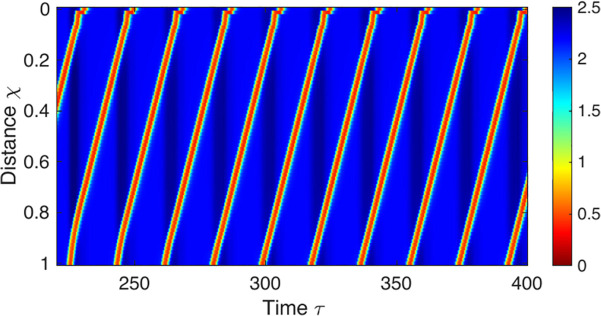
A repetitive retrograde contractions pattern obtained through the neuromechanical model by disabling excitation of inhibitory neuronal population through mechanoreceptors (*S*_*I*_). The figure displays the spatio-temporal topography of the non-dimensional color-coded cross-sectional area with respect to *A*_*o*_. χ is non-dimensional length with respect to *L* and τ is non-dimensional time with respect to $\tau_{E}$. The parametric values used to obtain these results are listed in Table K in S3 Text.

Repetitive retrograde contractions emerge with decreased excitation of inhibitory neurons via mechanoreceptors (*S*_*I*_) or increased excitation of excitatory neurons via mechanoreceptors (*S*_*E*_) (Fig 8). The retrograde pattern observed through our model is a result of a phase shift in the excitatory signal, opposing the antegrade pattern. Importantly, this phenomenon is not due to a retrograde traveling signal, as the excitatory and inhibitory pathways remain unidirectional. Further details on reversing propagation direction are discussed extensively in our recent work [53].

## Discussion

This work closes the gap in clarifying the connection between aberrant neural circuitry and emergent mechanical dysfunction. The organ-scale model provides a functional framework for exploring how interactions between excitatory and inhibitory neuronal populations shape muscle dynamics, without assuming detailed or fully mapped neural pathways. The main objectives were to unveil the underlying mechanism of RACs by providing a neural circuitry that reproduces this pattern and to elucidate the mechanisms that lead to neurologically driven EMDs. Our results suggest that abnormal contraction patterns may emerge from disruptions in the relative activation of excitatory and inhibitory neurons, consistent with prior hypotheses regarding the pathophysiology of esophageal motility disorders [43,44,65]. Additionally, we addressed open questions about the emergence of RACs [42]. By examining scenarios in which distension-sensitive mechanoreceptors were disabled, we demonstrated that RACs are locally triggered, supporting the notion that they represent a form of secondary peristalsis. This showcases how neuromechanical models can bring clarity to clinical observations.

An exact mapping of the neural circuits that coordinate the diverse motor patterns observed in esophageal distension studies is not available. Previous works attempting to define the underlying neurological mechanism are phenomenological. Here, we provide a conceptually simple mathematical model that is based on these phenomenological studies to investigate the mechanisms by which the neural circuit can produce the array of esophageal contraction patterns.

While it is possible to model an organ at various levels of detail, from the molecular to the system-wide, our aim is to strike a balance that is most useful for clinical and diagnostic applications. Detailed models, though valuable, may not always offer practical insights for physicians. Our system-level model seeks to formalize this intuitive understanding, providing a mathematical framework that can identify key mechanisms underlying observed behaviors. This, in turn, can guide further exploration into more detailed models.

It is essential to acknowledge certain limitations. First, physiological properties such as axonal conduction velocities, synaptic delays, durations of junctional potentials (excitatory and inhibitory) and synaptic potentials in neurons have been shown to play important roles in the generation and timing of propagating motor activity in the gastrointestinal tract [9,66–68]. Their underlying mechanisms therefore represent potential sites for dysfunction leading to esophageal dysmotility. These mechanisms are not included in the current model.

Second, the model does not distinguish between intrinsic afferent neurons (within the esophageal wall) and extrinsic afferent pathways (vagal and spinal). The omission of this distinction may limit the model’s ability to reveal how different afferent sources contribute separately to normal and disordered motility.

Third, the model only explicitly includes the contribution of excitatory motor neurons to the muscle contraction pattern. This is despite evidence of inhibitory motor neurons in the esophageal neural circuitry [69,70]. While a future version could incorporate inhibitory motor neurons acting directly on muscle, our findings demonstrate that key characteristics of esophageal distension tests can be captured without it.

Fourth, the model does not include myogenic mechanisms of smooth-muscle contraction. In the lower esophageal sphincter, myogenic control is well established [14,71,72]. For the esophageal body, although less clear, in vitro work in cat esophagus showed that peristalsis can be generated purely myogenically under specific conditions [73] and work in opossum suggested propagated contractions may occur independently of extrinsic input [74,75]. These findings imply that myogenic mechanisms may provide an additional layer of control. However, their role is not represented in the current model and warrants further study.

Fifth, our parameter values, while effective in reproducing observed behaviors, still require calibration. Extracting those values experimentally is challenging [26], especially considering the current lack of a fully explained and quantified esophageal neuronal pattern. As such, we relied on qualitative clinical observations reported in the literature to guide our model parameters. Future work should aim for direct experimental validation of these parameters.

Advancing towards experimental validation can be achieved through additional pharmacological studies, electrophysiological recordings, and by studying the neural circuitry at the molecular level through immunohistochemical, optogenetics, neuronal tracing, and calcium imaging [12,22,23,70,76–79]. A recent study on the peristaltic reflex of the colon provides a promising template, revealing the physiological and neural elements involved using multiple experimental methods [79].

Lastly, our model excludes the lower esophageal sphincter. Repetitive retrograde contractions have previously been theorized to arise from either impaired inhibitory innervation or esophageal outflow obstruction [43]. A recent clinical study supports the former hypothesis, attributing repetitive retrograde contractions to neural imbalance leading to excess excitation [43]. Our investigation aligns with the neural-imbalance hypothesis, but does not exclude the outflow-obstruction mechanism. Incorporating a lower esophageal sphincter model would enable exploration of such alternative pathways in future studies.

Despite these limitations, this work opens a new area of investigation in esophagology that is based on mechanophysiology (mechanics-based organ function). One of the leading diagnostic protocols for EMDs is the Chicago Classification scheme [35]. This work is a milestone in our effort to develop the first mechanics-guided disease classification and diagnostic protocol for EMDs [45,80], which is an ongoing aspirational goal.

Organ-scale neuromechanical models can mark a significant step forward in bridging the gap between clinical observations and mechanistic understanding, thereby aiding the development of effective neurologically focused treatment approaches. More broadly, the present work establishes a template to interrogate neurologically driven mechanophysiological pathologies of other organs.

## Methods

### Overview of mathematical model

In this section, we provide a brief overview of esophageal neuromechanics and outline how its various components are represented in our empirically guided model. We refer to it as ‘empirically guided’ because the inclusion of specific neural and mechanical elements is mostly informed by clinical phenomenological experiments. The mathematical details of the model are discussed in the following sections.

The model focuses on the smooth muscle region of the esophagus, which underlies the response observed during FLIP testing. In this region, peristalsis can occur independently of central input and is primarily mediated by intrinsic enteric neural circuits that respond to local distension through mechanosensory feedback [10,33]. Thus, the model is constructed to represent the local, ENS-driven control of smooth muscle peristalsis without explicitly including extrinsic vagal or spinal pathways.

The esophagus undergoing FLIP testing is modeled as a closed-end, flexible tube filled with an incompressible, viscous fluid [81]. The circular muscle layer is modeled as the primary contractile element responsible for lumen closure and bolus propulsion. The longitudinal muscle layer is not explicitly included, as previous computational work demonstrated that circular muscle contraction alone is sufficient to generate effective bolus transport, whereas longitudinal muscle shortening alone cannot propel the bolus [82].

The neural circuitry is represented as a chain of coupled relaxation oscillators (Fig 2), a framework widely used to describe oscillatory or wave-like activity in biological systems, including central pattern generators [83], slow waves in the stomach [84], and intestines [24,85–88].

The coupling between the neuronal model and the body mechanics is modeled as follows. Since the esophageal response to FLIP is known to be involuntary and assumed to be triggered locally through distension-sensitive mechanoreceptors [39,42,89], the model is such that excitation of the circuit is led by local feedback. The neural circuitry receives excitatory signals from mechanoreceptors along the esophageal body (Fig 2A). In return, when the amount of local excitation rises above a given threshold, excitatory motor neurons cause a contraction of esophageal muscles [12,90]. The contraction wave is coordinated by a sequential excitation [32,58]. The innervation pathways for each neuronal population (excitatory and inhibitory) are discussed next.

**Excitatory input from mechanoreceptors.** When the FLIP bag inside the smooth muscle section of the esophagus is inflated, it causes the esophagus to distend, activating the proprioceptive channel that responds to changes in strain (deformation) [20,89]. These mechanoreceptors are part of the esophageal neural circuit and are distributed along the length of the esophagus. Thus, in our neuronal model, mechanoreceptors are activated when they sense that local strain increases above a threshold and send excitatory signals to both excitatory and inhibitory neuronal populations.

Since the FLIP records only from the smooth muscle portion of the esophagus, the present model focuses on ENS-driven mechanosensory feedback and does not explicitly include extrinsic vagal or spinal pathways. Thus, the model presumes all mechanoreceptors are entirely intrinsic to the ENS.

Since the esophagus contracts proximal to a bolus and relaxes distal to a bolus [31], activated mechanoreceptors are set to send excitatory signals to rostral excitatory populations and caudal inhibitory populations [10]. Additionally, it has been reported that during esophageal distension, the portion of the esophageal body that is located distal to the distended balloon typically remains inactive or quiescent [62]. The esophagus distal to the excited portion is inhibited to accommodate for the incoming bolus [10,31]. Therefore, the input from the distension-sensitive mechanoreceptors to the inhibitory neuronal population affects all inhibitory neurons caudal to the sensed distension. The input from mechanoreceptors to the excitatory neuronal population is more local, such that sensed distension only inputs an excitatory signal to rostral excitatory neurons that are within 10% of esophageal length.

Note that while our model includes only intrinsic mechanoreceptors responsive to distension, these can be conceptualized as components of a broader mechanosensory network that also includes extrinsic vagal and spinal afferents.

**Input from adjacent neuronal populations.**

Compared with the small intestine and colon, the organization of intrinsic interneuron-like elements in the esophageal myenteric plexus is less well defined in the literature [9,22,66,91,92]. Nevertheless, descending neural pathways have been described in which neurons project orally to synapse onto cholinergic excitatory motor neurons, thereby initiating contraction in response to esophageal distention [93]. In addition, depolarization of one muscle cell in the esophagus can induce electrotonic depolarization of neighboring cells in an aboral direction, contributing to the coordinated propagation of motor activity [32,94].

To capture these coordinated effects with a compact segmental model, nearest-neighbor couplings are introduced as phenomenological reductions of multiple biological processes [95–97]. A unidirectional aboral excitatory coupling between excitatory populations of adjacent segments (parameter *b* in Fig 2A) reproduces the dominant directionality of esophageal propagation and abstracts the combined effects of descending excitatory projections. A directed inhibitory coupling from the inhibitory population in one segment to the caudal excitatory population (parameter *d* in Fig 2A) captures the functional consequence of inhibitory reflex signaling that shapes wavefront timing and spatial patterning.

**Intrasegmental synaptic inputs.** Within each segment, local interactions between excitatory and inhibitory neuronal populations determine the overall excitatory drive to motor output. In the model, these interactions are represented by a set of coupling terms (parameters *a*, *c*, *e*, and *f* in Fig 2A). These are trivial elements that capture how activity within a segment regulates its own excitability and responsiveness, and are often used when modeling the electrical activity using a phenomenological approach [87,95,98]. They are not intended to reproduce detailed synaptic anatomy.

Note that there is no direct evidence for direct inhibitory synapses within the esophageal myenteric plexus. However, functional inhibitory signaling in the esophagus is well documented and is important for coordinating contraction-relaxation sequences [14,93]. In the model, this inhibitory influence is represented as an inhibitory input to the excitatory motor population, which effectively produces muscle relaxation by suppressing excitatory drive rather than by introducing an explicit inhibitory motor-to-muscle projection.

Table A in S2 Text provides additional information on the neural circuit.

### Body mechanics and fluid equations

The flow inside the FLIP device placed in the esophageal lumen is modeled as a one-dimensional, fluid-filled, flexible tube that is closed on both ends [81,99,100]. The conservation of mass and momentum equations are

$$\frac{\partial A}{\partial t}+\frac{\partial \left(Au\right)}{\partial x}=0,$$ (1)

and

$$\frac{\partial u}{\partial t}+u\frac{\partial u}{\partial x}=-\frac{1}{\rho }\frac{\partial P}{\partial x}-\frac{8\pi \mu u}{\rho A},$$ (2)

respectively. A parabolic flow is assumed everywhere. In the equations above, *A*(*x*,*t*), *u*(*x*,*t*), *P*(*x*,*t*), ρ and μ are the tube cross-sectional area, fluid velocity (averaged at each cross-sectional area), pressure inside the tube, fluid density, and fluid viscosity, respectively. We introduce a linear constitutive relation

$$P=K_{e}\left(\frac{A}{A_{o}\theta }-1\right)+P_{o}.$$ (3)

to complete the system [101,102]. Here, *P*_*o*_, *K*_*e*_, and *A*_*o*_ are external pressure, tube stiffness, and undeformed reference area (cross-sectional area of the tube when *P* = *P*_*o*_), respectively. Lastly, the neurally controlled contraction of the esophageal lumen is set through dynamically varying the rest cross-sectional area of the tube by a factor θ(x,t) [103,104]. θ(x,t) captures the muscular dynamics, induced by the neural activity discussed next.

### Neural circuitry: excitation, inhibition, and muscle contraction pattern

To represent the electrical activity of the entire organ, we introduce a system of locally coupled Wilson–Cowan oscillators, distributed uniformly along the length of the esophagus. Each unit consists of two neuronal populations, excitatory (*E*) and inhibitory (*I*) (Fig 2A). The values of *E*_*i*_(*t*) and *I*_*i*_(*t*) at each oscillator depict the activity levels of the excitatory and inhibitory neuron populations. The differential equations for the time-dependent variation of averaged excitatory and inhibitory neuronal activities at node *i* introduced by [51] with the unidirectional coupling introduced by [97] are

$$\tau_{E}{\dot{E}}_{i}=-E_{i}+(1-E_{i})\sigma_{E}[aE_{i}+bE_{i-1}-eI_{i}-dI_{i-1}+S_{E,i}],$$ (4)

and

$$\tau_{I}{\dot{I}}_{i}=-I_{i}+(1-I_{i})\sigma_{I}[cE_{i}-fI_{i}+S_{I,i}].$$ (5)

The intrasegmental connectivity parameters *a*, *e*, *c*, and *f* represent the average synaptic weight of excitatory and inhibitory synapses per cell in the excitatory or inhibitory population. The connectivity coefficient *b* denotes the unidirectional excitatory connections between the excitatory populations of neighboring segments (excitatory signal from nearest posterior excitatory population). The connectivity coefficient *d* signifies the unidirectional inhibitory connections from a segment’s inhibitory population to the nearest anterior segment’s excitatory population. For the relaxation oscillators located at the rostral end, *b* = *d* = 0. The time constants $\tau_{E}$ and $\tau_{I}$ dictate the decay of the excitatory and inhibitory activities, and determine the timescales of the activities. The sigmoid function characterizes the switching threshold defined as

$$\sigma_{E/I}[x]=\frac{1}{1+\text{exp}[-\lambda_{E/I}(x-\phi_{E/I})]}-\frac{1}{1+\text{exp}(\lambda_{E/I}\phi_{E/I})}.$$ (6)

where λ is activation speed (slope of sigmoid), and ϕ is activation threshold (location of sigmoid’s maximum slope) [96,97]. Lastly, *S*_*E*,*i*_ and *S*_*I*,*i*_ symbolize the local excitatory inputs to each population at oscillator *i*, capturing the mechano-sensory feedback coming from the distension-sensitive mechanoreceptors [14,15], discussed next.

The distension-sensitive mechanoreceptors are activated when they sense that local deformations increase above a threshold ($\hat{\alpha }$). In our model, it is equivalent to pressure or hoop stress dependent trigger defined by $\left(A(x,t)/A_{o}\theta (x,t)-\hat{\alpha }\right)$. By including θ, we ensure that the receptors sense strain relative to an updated reference area $\left(A_{o}\theta (x,t)\right)$ rather than the rest reference area (*A*_*o*_). As discussed in a previous section, when activated, a mechanosensory receptor sends excitatory signals to rostral excitatory populations and excitatory signals to caudal inhibitory populations. The input to excitatory neurons from distension-sensitive mechanoreceptors is more local, meaning that distension excites excitatory neurons just proximal of the activated receptor. On the other hand, the input from distension-sensitive mechanoreceptors to inhibitory neurons is applied to all inhibitory neurons caudal to the activated receptor. Thus, *S*_*E*_ and *S*_*I*_ input for a segment located in *x*_*i*_ are defined as

$$S_{E,i}=w_{E}\sigma_{S}[\int_{x_{i}}^{L}h(y)\beta_{E}(x_{i}-y)\;dy]\;\text{and}\;S_{I,i}=w_{I}\sigma_{S}[\int_{0}^{x_{i}}h(y)\beta_{I}(x_{i}-y)\;dy],$$ (7)

where $\sigma_{S}[x]=\text{tanh}[g_{S}x]$, *w*_*E*_ and *w*_*I*_ are the strength of the net sensory feedback coming from the distension-sensitive mechanoreceptors receptors, $\beta_{E}(x)=0.5+0.5\text{tanh}(g_{E}(x+x_{s}))$, $\beta_{I}=1$, $h(x)=max[A(x,t)/A_{o}\theta (x,t)-\hat{\alpha },0]$, *L* is esophageal length, *g*_*S*_ and *g*_*E*_ are gains, and *x*_*s*_ is horizontal shift. The schematic of the distension-induced excitation is presented in Fig 2B.

When the excitatory neural activity surpasses a certain threshold, it excites the smooth muscles, causing them to contract. The coupling between the neural circuit and the mechanical model is done through solving for the activation function θ, which receives input from excitatory neuronal population *E*, such that

$$\tau_{\theta }{\dot{\theta }}_{i}=1-\theta_{i}-\sigma_{\theta }[E_{i}-\hat{E}].$$ (8)

In this equation, $\tau_{\theta }$ is a time constant, $\hat{E}$ the excitatory threshold for muscle activation, and $\sigma_{\theta }[x]=0.5[1-\theta_{o}+(1-\theta_{o})\text{tanh}(g_{\theta }x)]$, where $g_{\theta }$ is the gain, and $\theta_{o}$ is the maximum contraction strength.

In the muscle pattern equation above, only excitatory motor pathways are represented, providing a minimal framework to examine how mechanosensory feedback within the ENS can generate and sustain secondary peristaltic activity. Incorporating inhibitory motor pathways, as previously done in [24] and [85], would allow for more complete reflex dynamics, but is beyond the present scope.

### Non-dimensional form

The dynamic system of equations described above is non-dimensionalized using

$$A=\alpha A_{o},\;t=\tau \tau_{E},\;u=UL/\tau_{E},\;P=pK_{e},\;\text{and}\;x=\chi L,$$ (9)

where α, τ, *U*, *p*, and χ are the non-dimensional variables for cross-sectional area, time, velocity, pressure, and position, respectively [81,99]. Thus, dimensional Eqs ((1), (2), (3), (4), (5), and (8)) become

$$\frac{\partial \alpha }{\partial \tau }+\frac{\partial \left(\alpha U\right)}{\partial \chi }=0,$$ (10)

$$\frac{\partial U}{\partial \tau }+U\frac{\partial U}{\partial \chi }+\psi \frac{\partial p}{\partial \chi }+\beta \frac{U}{\alpha }=0,$$ (11)

$$p=\left(\frac{\alpha }{\theta }-1\right),$$ (12)

$${\dot{E}}_{i}=-E_{i}+(1-E_{i})\sigma_{E}[aE_{i}+bE_{i-1}-eI_{i}-dI_{i-1}+S_{E,i}],$$ (13)

$${\hat{\tau }}_{I}{\dot{I}}_{i}=-I_{i}+(1-I_{i})\sigma_{I}[cE_{i}-fI_{i}+S_{I,i}]\;\text{and}$$ (14)

$${\hat{\tau }}_{\theta }{\dot{\theta }}_{i}=1-\theta_{i}-\sigma_{\theta }[E_{i}-\hat{E}],$$ (15)

respectively, where $\psi =K_{e}\tau_{E}^{2}/\rho L^{2}$, $\beta =8\pi \mu L\tau_{E}/\rho A_{o}$, ${\hat{\tau }}_{\theta }=\tau_{\theta }/\tau_{E}$, and ${\hat{\tau }}_{I}=\tau_{I}/\tau_{E}$.

### Initial and boundary conditions

The fluid in the tube is initially at rest and the cross-sectional area is uniform, hence

$$U\left(\chi ,\tau =0\right)=0\;\text{and}\;\alpha \left(\chi ,\tau =0\right)=S_{\text{IC}}\theta (\chi ,\tau =0),$$ (16)

where $S_{\text{IC}}=\text{Volume}/A_{o}$ [100,105]. Additionally,

$$E_{\tau =0}=0,\;\;I_{\tau =0}=0,\;\text{and}\;\theta_{\tau =0}=1,$$ (17)

since the neuronal system is at rest. Lastly, recall that the tube is closed on both ends, thus,

U(χ=0,τ)=0andU(χ=1,τ)=0. (18)

To obtain a boundary condition for α, we plug Eq (12) and the velocity boundary condition in Eq (18) into Eq (11), which yields

$${\frac{\partial }{\partial \chi }\left(\frac{\alpha }{\theta }\right)|}_{\chi =0,\tau }=0\;and\;{\frac{\partial }{\partial \chi }\left(\frac{\alpha }{\theta }\right)|}_{\chi =1,\tau }=0.$$ (19)

### Numerical implementation

The system of equations is solved using MATLAB ode45 function for the time derivatives and central difference discretization for the spatial derivatives. The discrete expressions for the external source terms in Eq (7) are

$$S_{E,i}=w_{E}\sigma_{S}[\sum_{k=i}^{N}max(\alpha_{k}/\theta_{k}-\hat{\alpha },0)\beta_{E}(\chi_{i}-\chi_{k})\;\Delta \chi ],\;\text{and}\;\;S_{I,i}=w_{I}\sigma_{S}[\sum_{k=1}^{i}max(\alpha_{k}/\theta_{k}-\hat{\alpha },0)\;\Delta \chi ],$$ (20)

where *N* denotes the number of nodes.

Table 1 lists the model’s parameters and their values. Values for the parameters defining esophageal body mechanics and fluid properties are approximated based on clinical data and previous computational studies [81,82,99,102]. The parameters involved in the neural equations are chosen such that the system has a single unstable fixed point and a stable limit cycle in response to the constant external stimulus *S*_*E*_ and *S*_*I*_. Depending on the values of the parameters in the system, one can obtain multiple equilibria with different stability properties. For simplicity, we choose the values introduced by [51] with small variations. For example, theoretical work concerning Wilson-Cowan oscillators often sets *S*_*I*_ = 0 for simplicity [51]. By setting $S_{I}\neq 0$, we ensure that both excitatory and inhibitory populations “turn-on” when distension is applied, independent of one another. A theoretical overview of relaxation oscillators, limit cycle solution, and the constraints introduced by modeling esophageal response is provided in the S1 Text.

**Table 1:** List of parameters and their values.

Parameter	Value	Parameter	Value	Parameter	Value
ψ	3000	β	100	$\theta_{o}$	0.05
$S_{\text{IC}}$	2	$\hat{\alpha }$	1.5	*x*_*s*_	0.1
${\hat{\tau }}_{\theta }$	0.2	${\hat{\tau }}_{I}$	4	*a*	16
*b*	20	*c*	12	*d*	40
*e*	15	*f*	3	*w*_*E*_	1.6
*w*_*I*_	1.35	$\phi_{E}$	4	$\phi_{I}$	3.7
$\lambda_{E}$	1.3	$\lambda_{I}$	2	*g*_*S*_	1000
*g*_*E*_	1000	$g_{\theta }$	5	$\hat{E}$	0.3
*N*	70				

## Supporting information

S1 Text Background and theory - relaxation oscillators and limit cycle solution. **Fig A. Plots of the state variables (*E* and *I*) over time for relaxation oscillator with different inputs.** **(a)** No input is introduced (*S*_*E*_ = 0), so the system remains at rest, where *E* and *I* do not change over time, remaining at zero. **(b)** System’s response to short-term stimuli creating excitable regime, in which *E* and *I* increase in response to the transient stimuli before decaying to rest values. **(c)** System’s response to sustained stimuli, creating a limit cycle solution, in which *E* and *I* fluctuate at constant pattern over time.
**Fig B. Phase diagram of a single Wilson-Cowan oscillator.** The oscillator is defined by Eqs (4) and (5), and the parameters in Table 1 in the main manuscript, with *b* = *d* = 0. The diagram includes three nullclines, one for *I* (black) and two for *E* nullclines (blue *S*_*E*_ = 0 and red *S*_*E*_ = 1.6). For *S*_*E*_ = 1.6, a limit cycle solution emerges (Fig A in S1 Text).
(PDF)

S2 Text Empirically guided table. **Table A.** Description of mathematical terms and associated literature.
(PDF)

S3 Text Parametric values for simulations in the main manuscript. **Table A.** List of parameters and their values used to obtain the results in Figs 4A and 4B of the main manuscript.
**Table B.** List of parameters and their values used to obtain the results in Fig 4C of the main manuscript.
**Table C.** List of parameters and their values used to obtain the results in Fig 4D of the main manuscript.
**Table D.** List of parameters and their values used to obtain the results in Fig 4E of the main manuscript.
**Table E.** List of parameters and their values used to obtain the results in Fig 4F of the main manuscript.
**Table F.** List of parameters and their values used to obtain the results in Fig 5A of the main manuscript.
**Table G.** List of parameters and their values used to obtain the results in Fig 5B of the main manuscript.
**Table H.** List of parameters and their values used to obtain the results in Fig 6A of the main manuscript.
**Table I.** List of parameters and their values used to obtain the results in Fig 6B of the main manuscript.
**Table J.** List of parameters and their values used to obtain sustained panesophageal contraction.
**Table K.** List of parameters and their values used to obtain the results in Fig 8 of the main manuscript.
(PDF)

S4 TextSensitivity testing and robustness analysis.**Fig A. Solution of sensitivity test to varying the value of parameter *e***. **(a)** Color-coded topography of muscle contraction pattern (θ) with the parameter *e* modified to 0.7*e*, representing a 30% decrease from the original value of *e*. **(b)** Color-coded topography of muscle contraction pattern (θ) with the parameter *e* modified to 1.2*e*, representing a 20% increase from the original value of *e*. **(c)** Plot of the intersegmental phase lag as a function of percentage deviation from the baseline value of parameter *e* where 0% corresponds to the baseline *e* value (*e* = 15).**Fig B. Solution of sensitivity test to varying the value of parameter *f***. **(a)** Color-coded topography of muscle contraction pattern (θ) with the parameter *f* modified to 0.5*f*, representing a 50% decrease from the original value of *f*. **(b)** Color-coded topography of muscle contraction pattern (θ) with the parameter *f* modified to 2*f*, representing a 100% increase from the original value of *f*. **(c)** Plot of the intersegmental phase lag as a function of percentage deviation from the baseline value of parameter *f* where 0% corresponds to the baseline *f* value (*f* = 3).**Fig C. Solution of sensitivity test to varying the value of parameter *w*_*I*_**. **(a)** Color-coded topography of muscle contraction pattern (θ) with the parameter *w*_*I*_ modified to 0.5*w*_*I*_, representing a 50% decrease from the original value of *w*_*I*_. **(b)** Color-coded topography of muscle contraction pattern (θ) with the parameter *w*_*I*_ modified to 1.2*w*_*I*_, representing a 20% increase from the original value of *w*_*I*_. **(c)** Plot of the contraction propagating speed as a function of percentage deviation from the baseline value of parameter *w*_*I*_ where 0% corresponds to the baseline *w*_*I*_ value (*w*_*I*_ = 1.35).**Fig D. Solution of sensitivity test to varying the value of parameter *b***. **(a)** Plot of the intersegmental phase lag as a function of percentage deviation from the baseline value of parameter *b* where 0% corresponds to the baseline *b* value (*b* = 20). **(b)** Plot of the segment activity duration (how long an oscillator is active) as a function of percentage deviation from the baseline value of parameter *b*. Due to the relation between muscle contraction pattern (θ) and the excitatory activity level (*E*) displayed in Eq (8) in the main manuscript, segment activity duration is directly related to contraction strength. **(c)** Color-coded topography of muscle contraction pattern (θ) with the parameter *b* modified to 0.7*b*, representing a 30% decrease from the original value of *b*. **(d)** Color-coded topography of muscle contraction pattern (θ) with the parameter *b* modified to 2*b*, representing a 100% increase from the original value of *b*.**Fig E. Solution of sensitivity test to varying the value of parameter *d***. **(a)** Color-coded topography of muscle contraction pattern (θ) with the parameter *d* modified to 0.5*d*, representing a 50% decrease from the original value of *d*. **(b)** Color-coded topography of muscle contraction pattern (θ) with the parameter *d* modified to 1.5*d*, representing a 50% increase from the original value of *d*.**Fig F. Color-coded muscle contraction pattern (θ) topography obtained by the neuromechanical model with randomly sample values from a Gaussian distribution with specific mean and standard deviation**. **(a)** Irregularities introduced to *c* with mean=12 and standard deviation = 3. **(b)** Irregularities introduced to *e* with mean=15 and standard deviation = 10. **(b)** Irregularities introduced to *d* with mean=40 and standard deviation = 20. **(d)** Irregularities introduced to *b* with mean=20 and standard deviation = 10.(PDF)

S5 Text Numerical origin of the apparent discontinuity in simulated CSA. (PDF)
