# Supplementary material for: Modeling based insights into mechanical dysfunction in esophageal motility disorders
Source: PLoS Comput Biol. 2025 Dec 26;21(12):e1013778. doi: 10.1371/journal.pcbi.1013778 (PMC12779157; doi:10.1371/journal.pcbi.1013778)
Supplement: S1 Text — Fig A. Plots of the state variables (E and I) over time for relaxation oscillator with different inputs. (a) No input is introduced (SE = 0), so the system remains at rest, where E and I do not change over time, remaining at zero. (b) System’s response to short-term stimuli creating excitable regime, in which E and I increase in response to the transient stimuli before decaying to rest values. (c) System’s response to sustained stimuli, creating a limit cycle solution, in which E and I fluctuate at constant pattern over time. Fig B. Phase diagram of a single Wilson-Cowan oscillator. The oscillator is defined by Eqs (4) and (5), and the parameters in Table 1 in the main manuscript, with b = d = 0. The diagram includes three nullclines, one for I (black) and two for E nullclines (blue SE = 0 and red SE = 1.6). For SE = 1.6, a limit cycle solution emerges (Fig A in S1 Text). (PDF) [file pcbi.1013778.s001.pdf]

S1 Text. Background and theory - relaxation oscillators and limit cycle solution

A relaxation oscillator is a mathematical model of electronic circuit that can exhibit a variety of behaviors that depend on the intrinsic and input parameters defining it [1]. Relaxation oscillators have been used for years to model the electrical activity of the heart due to their ability to display rhythmic, repetitive patterns known as limit cycles [2–4]. The shape, frequency, and amplitude of the oscillations depend on the properties of the oscillator [1].

Unlike the heart, the esophagus does not exhibit a consistent rhythmic pattern in its natural state. In the absence of any electrical input or stimulation, the esophagus remains at rest [5]. When subjected to short-term stimuli, the esophagus briefly becomes active, undergoing a single cycle of activity before gradually returning to its resting state [6]. Lastly, under sustained stimuli (through sustained volumetric distension), a rhythmic pattern emerges in its behavior [7]. By definition, relaxation oscillators can exhibit these distinct behaviors based on the presence and nature of external inputs, as discussed next.

In the absence of any prescribed input, a relaxation oscillator remains in a resting state. During this time, the system's output remains constant (Fig Aa). When a transient input is introduced, a relaxation oscillator responds by producing a transient and distinguishable output. Following the excitation, the system gradually decays to its rest state (Fig Ab). Lastly, if the input is sustained and exceeds a certain threshold, the relaxation oscillator can transition into a self-sustained, rhythmic oscillatory behavior known as a limit cycle solution (Fig Ac).

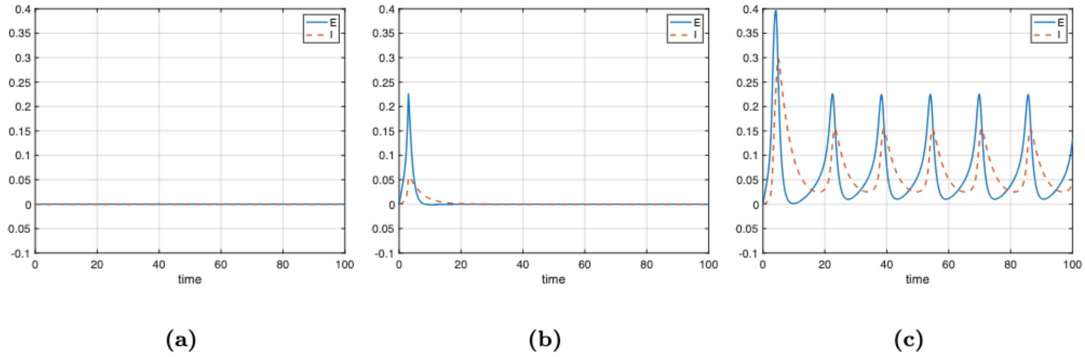

Fig A. **Plots of the state variables (E and I) over time for relaxation oscillator with different inputs.** **(a)** No input is introduced ($S_E = 0$), so the system remains at rest, where E and I do not change over time, remaining at zero. **(b)** System's response to short-term stimuli creating excitable regime, in which E and I increase in response to the transient stimuli before decaying to rest values. **(c)** System's response to sustained stimuli, creating a limit cycle solution, in which E and I fluctuate at constant pattern over time.

Since this work is mostly concerned with esophageal rhythmic response, we wish to further explain limit cycle oscillations. To understand the emergence of limit cycle oscillations, we employ phase analysis, displayed in Fig B. Through this approach, we can distinctly identify the threshold value that the input from stretch must exceed to transition from the resting state (Fig Aa) to the limit cycle oscillations (Fig Ac).

In Fig B, the nullclines ($dE/dt = 0$ and $dI/dt = 0$) of a relaxation oscillator are plotted. A nullcline represents the trajectory at which the derivative of a variable becomes zero [8]. Here, E and I denote the state variables, where E is termed as the fast variable and I as the slow variable. This nomenclature stems from their respective rates of change; E changes rapidly compared to I, hence termed as fast, while I changes more gradually, hence termed as slow. The axes in Fig B represent these state variables. The plot illustrates that as the E nullcline ($dE/dt = 0$) shifts upwards, the intersection of the nullclines shifts, eventually intersecting somewhere along the middle branch of the E nullcline. Consequently, a limit cycle solution emerges [8]. For additional information on dynamic systems, refer to [1, 9, 10].

A chain of limit cycle oscillators is a well-studied system [11, 12]. The oscillators in such a system are coupled, often identical, and each oscillator exhibits a limit cycle solution, independent of the coupling [13–15]. Such systems often exhibit "frequency pulling", where all oscillators converge into an entrained frequency. However, they are not synchronized in phase, as there is phase shift between adjacent oscillators [11, 16]. At steady state, the oscillators phase lock, meaning that they oscillate at constant phase for all subsequent oscillations, creating a special case of synchrony [17, 18]. This emerging pattern, known to be the

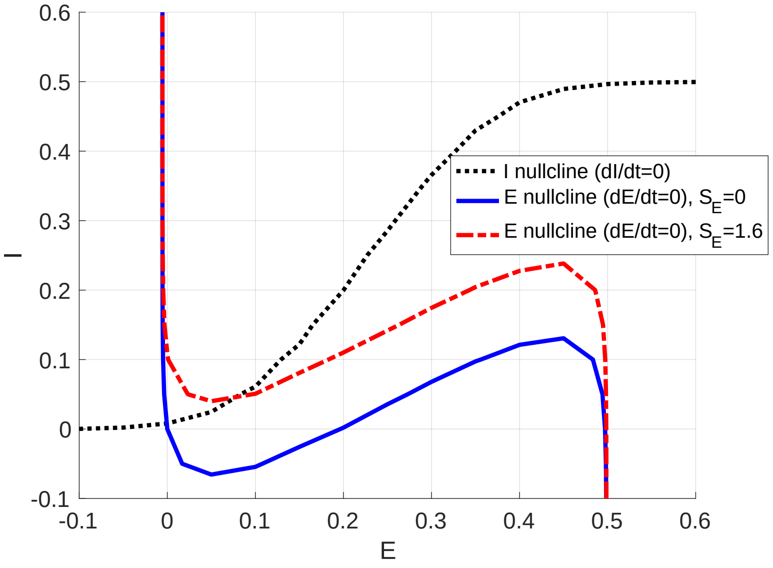

Fig B. **Phase diagram of a single Wilson-Cowan oscillator.** The oscillator is defined by Eqs. (4) and (5), and the parameters in Table 1 in the main manuscript, with $b = d = 0$. The diagram includes three nullclines, one for I (black) and two for E nullclines (blue $S_E = 0$ and red $S_E = 1.6$). For $S_E = 1.6$, a limit cycle solution emerges (Fig Ac).

fundamental solution of coupled system in equilibrium state, is the key element in creating the propagating attribute of the system [19, 20].

References

[1] DeLiang Wang. Relaxation oscillators and networks. *Wiley Encyclopedia of Electrical and Electronics Engineering*, 18:396–405, 1999.

[2] Natalia A. Trayanova, Jason Constantino, and Viatcheslav Gurev. Electromechanical models of the ventricles. *American Journal of Physiology-Heart and Circulatory Physiology*, 301(2):H279–H286, 2011.

[3] C Cherubini, S Filippi, P Nardinocchi, and L Teresi. An electromechanical model of cardiac tissue: Constitutive issues and electrophysiological effects. *Progress in Biophysics and Molecular Biology*, 97 (2-3):562–573, 2008.

[4] Peng Du, Greg O'Grady, John B. Davidson, Leo K. Cheng, and Andrew J. Pullan. Multiscale modeling of gastrointestinal electrophysiology and experimental validation. *Critical Reviews in Biomedical Engineering*, 38(3), 2010.

[5] Raj K. Goyal and Arun Chaudhury. Physiology of normal esophageal motility. *Journal of Clinical Gastroenterology*, 42(5):610, 2008.

[6] William G. Paterson, Satish Rattan, and Raj K. Goyal. Esophageal responses to transient and sustained esophageal distension. *American Journal of Physiology-Gastrointestinal and Liver Physiology*, 255(5): G587–G595, 1988.

[7] Dustin .A Carlson, Wenjun Kou, Melina Masihi, Shashank Acharya, Alexandra J. Baumann, Erica N. Donnan, Peter J. Kahrilas, and John E. Pandolfino. Repetitive antegrade contraction: a novel response to sustained esophageal distension is modulated by cholinergic influence. *American Journal of Physiology-Gastrointestinal and Liver Physiology*, 319(6):G696–G702, 2020. doi: 10.1152/ajpgi.00305.2020.

[8] Eugene M Izhikevich. *Dynamical systems in neuroscience.* MIT press, 2007.

[9] Roy M. Smeal, G. Bard Ermentrout, and John A. White. Phase-response curves and synchronized neural networks. *Philosophical Transactions of the Royal Society B: Biological Sciences*, 365(1551): 2407–2422, 2010.

[10] Johan Grasman. *Asymptotic methods for relaxation oscillations and applications*, volume 63. Springer Science & Business Media, 2012.

[11] E.E. Daniel, Berj L. Bardakjian, Jan D. Huizinga, and N.E. Diamant. Relaxation oscillator and core conductor models are needed for understanding of gi electrical activities. *American Journal of Physiology-Gastrointestinal and Liver Physiology*, 266(3):G339–G349, 1994.

[12] Stephanie R. Jones and Nancy Kopell. Local network parameters can affect inter-network phase lags in central pattern generators. *Journal of Mathematical Biology*, 52:115–140, 2006.

[13] Avis H. Cohen, G. Bard Ermentrout, Tim Kiemel, Nancy Kopell, Karen A. Sigvardt, and Thelma L. Williams. Modelling of intersegmental coordination in the lamprey central pattern generator for locomotion. *Trends in Neurosciences*, 15(11):434–438, 1992.

[14] James J. Collins and Ian N. Stewart. Coupled nonlinear oscillators and the symmetries of animal gaits. *Journal of Nonlinear Science*, 3:349–392, 1993.

[15] Eugene M. Izhikevich. Phase equations for relaxation oscillators. *SIAM Journal on Applied Mathematics*, 60(5):1789–1804, 2000.

[16] DeLiang Wang. Emergent synchrony in locally coupled neural oscillators. *IEEE Transactions on Neural Networks*, 6(4):941–948, 1995.

[17] Nancy Kopell and G. Bard Ermentrout. Symmetry and phaselocking in chains of weakly coupled oscillators. *Communications on Pure and Applied Mathematics*, 39(5):623–660, 1986.

[18] Shannon Campbell and DeLiang Wang. Synchronization and desynchronization in a network of locally coupled wilson-cowan oscillators. *IEEE Transactions on Neural Networks*, 7(3):541–554, 1996.

[19] Steven H. Strogatz and Ian Stewart. Coupled oscillators and biological synchronization. *Scientific American*, 269(6):102–109, 1993.

[20] Nancy Kopell and G. Bard Ermentrout. Chains of oscillators in motor and sensory systems, 2003.
