# Supplementary material for: Modeling based insights into mechanical dysfunction in esophageal motility disorders
Source: PLoS Comput Biol. 2025 Dec 26;21(12):e1013778. doi: 10.1371/journal.pcbi.1013778 (PMC12779157; doi:10.1371/journal.pcbi.1013778)
Supplement: S2 Text — Table A. Description of mathematical terms and associated literature. (PDF) [file pcbi.1013778.s002.pdf]

S2 Text. Empirically guided table

Our work aims to study emergent behaviors in the esophagus by abstracting the behavior of the organ as a whole, rather than focusing on the details of every physiological interaction. There is an inherent complexity in directly connecting the parameters in the phenomenological, system-level model developed in this work to the underlying physiological mechanisms of the neural circuit [1, 2]. This connection is a longstanding open question in the field of neuro- and electro-physiology modeling [1, 2]. One recent example is the ongoing work by neuroscientists studying *Drosophila* to reconcile the well-established central pattern generator model with the newly mapped connectome of the fly [3, 4].

Given this understanding, in this supplementary section, we demonstrate how the parameters in our model relate to the underlying physiology by linking each feature or element in the neural circuit to the experimental studies that justify these choices. This information is presented in Table A. Note that parameters a, c, e, and f are phenomenological coupling terms included to reproduce observed excitatory–inhibitory dynamics. They are not associated with specific identified synaptic pathways and therefore cite modeling precedents rather than physiological data.

Table A: Description of mathematical terms and associated literature

**Mathematical term**	**Parameter description**	**Associated literature**
E	Excitatory neuronal population. In this simplified model, E represents the aggregate activity of cholinergic excitatory motor neurons that drive contraction and transmit excitatory influence within a segment or between neighboring segments.	Existence and role of excitatory neurons: [5–8]
I	Inhibitory neuronal population. Represents the functional influence of inhibitory motor neurons that mediate relaxation and modulate excitatory activity to coordinate peristaltic activity.[1]	Existence and role of inhibitory neurons: [9–12]
S_E	Local excitatory input from mechanoreceptors to the excitatory population.	Existence of distension-induced mechanosensitive pathways in the esophagus: [13–15]
S_I	Excitatory input from mechanoreceptors to the inhibitory population.	Evidence for mechanosensitive activation and descending inhibition: [5, 11, 13, 16, 17]
b	Unidirectional, aboral coupling between excitatory populations of neighboring segments. Represents coordinated propagation of excitatory activity.[2]	Functional evidence of aborally propagating excitation: [8, 16, 18–20]. Phenomenological modeling precedents: [21, 22]
d	Unidirectional coupling from the inhibitory population of one segment to the excitatory population of the adjacent (aboral) segment. Represents functional descending inhibition that transiently suppresses excitation to shape wave propagation.[2]	Functional evidence of descending inhibition and inhibitory neurons: [11, 16, 18, 20]. Phenomenological modeling precedents: [21, 22]
a	Strength of local excitatory feedback within the excitatory population. Represents the net excitatory influence within a segment (not an explicit anatomical connection).[2]	Phenomenological modeling precedents: [21–28]
e	Strength of local inhibitory influence acting on the excitatory population. Represents the functional inhibitory modulation of excitatory neurons that underlies relaxation phases.[2]	Phenomenological modeling precedents: [21–28]
c	Strength of excitatory influence acting on the inhibitory population. Represents activation of inhibitory pathways following excitatory drive, consistent with sequential contraction–relaxation cycles.[2]	Phenomenological modeling precedents: [21–28]
f	Strength of inhibitory self-regulation within the inhibitory population. Represents a stabilizing local feedback maintaining the balance between excitation and inhibition.[2]	Phenomenological modeling precedents: [21–28]

1. Inhibitory motor neurons are known to play an essential role in esophageal peristalsis; in this model, their influence is represented by inhibitory populations that modulate excitatory drive rather than direct inhibitory motor-to-muscle projections [12, 18].
2. These parameters are phenomenological and do not represent explicit synaptic anatomy. They describe the net excitatory–inhibitory interactions within and between segments, consistent with similar modeling approaches.

References

[1] Jing Wui Yeoh, Alberto Corrias, and Martin L. Buist. Modelling human colonic smooth muscle cell electrophysiology. *Cellular and Molecular Bioengineering*, 10:186–197, 2017.

[2] Danielle L. Kurtin, Valentina Giunchiglia, Jakub Vohryzek, Joana Cabral, Anne C. Skeldon, and Ines R. Violante. Moving from phenomenological to predictive modelling: Progress and pitfalls of modelling brain stimulation in-silico. *Neuroimage*, 272:120042, 2023.

[3] Jasper S. Phelps, David Grant Colburn Hildebrand, Brett J. Graham, Aaron T. Kuan, Logan A. Thomas, Tri M. Nguyen, Julia Buhmann, Anthony W. Azevedo, Anne Sustar, Sweta Agrawal, Mingguan Liu, Brendan L. Shanny, Jan Funke, John C. Tuthill, and Wei-Chung Allen Lee. Reconstruction of motor control circuits in adult drosophila using automated transmission electron microscopy. *Cell*, 184(3):759–774, 2021.

[4] Victor Lobato-Rios, Shravan Tata Ramalingasetty, Pembe Gizem Özdil, Jonathan Arreguit, Auke Jan Ijspeert, and Pavan Ramdya. Neuromechfly, a neuromechanical model of adult drosophila melanogaster. *Nature Methods*, 19(5):620–627, 2022.

[5] Hyojin Park and Jeffrey L. Conklin. Neuromuscular control of esophageal peristalsis. *Current Gastroenterology Reports*, 1(3):186–197, 1999.

[6] SIMON JH Brookes, Bao Nan Chen, WENDY M Hodgson, and MARCELLO Costa. Characterization of excitatory and inhibitory motor neurons to the guinea pig lower esophageal sphincter. *GASTROENTEROLOGY-BALTIMORE THEN PHILADELPHIA-*, 111:108–117, 1996.

[7] Jyoti N. Sengupta. Electrophysiological recording from neurons controlling sensory and motor functions of the esophagus. *The American journal of medicine*, 111(8):169–173, 2001.

[8] Detlef Bieger and Winfried Neuhuber. Neural circuits and mediators regulating swallowing in the brainstem. *GI Motility online*, 2006.

[9] Haiheng Dong, Christopher W. Loomis, and Detlef Bieger. Distal and deglutitive inhibition in the rat esophagus: role of inhibitory neurotransmission in the nucleus tractus solitarii. *Gastroenterology*, 118 (2):328–336, 2000.

[10] Harold G. Preiksaitis, Louise Tremblay, and Nicholas E. Diamant. Nitric oxide mediates inhibitory nerve effects in human esophagus and lower esophageal sphincter. *Digestive diseases and sciences*, 39 (4):770–775, 1994.

[11] William G. Paterson and Indran B. Indrakrishnan. Descending peristaltic reflex in the opossum esophagus. *American Journal of Physiology-Gastrointestinal and Liver Physiology*, 269(2):G219–G224, 1995.

[12] Ravinder K. Mittal. Regulation and dysregulation of esophageal peristalsis by the integrated function of circular and longitudinal muscle layers in health and disease. *American Journal of Physiology-Gastrointestinal and Liver Physiology*, 311(3):G431–G443, 2016.

[13] Jyoti N. Sengupta. An overview of esophageal sensory receptors. *The American Journal of Medicine*, 108(4):87–89, 2000.

[14] Christina Brock, Hans Gregersen, C Prakash Gyawali, Christian Lottrup, Manuele Furnari, Edoardo Savarino, Luis Novais, Jens Brøndum Frøkjær, Serhat Bor, and Asbjørn Mohr Drewes. The sensory system of the esophagus—-what do we know? *Annals of the New York Academy of Sciences*, 1380(1): 91–103, 2016.

[15] Michael L. Frazure, Alyssa D. Brown, Clinton L. Greene, Kimberly E. Iceman, and Teresa Pitts. Rapid activation of esophageal mechanoreceptors alters the pharyngeal phase of swallow: Evidence for inspiratory activity during swallow. *Plos one*, 16(4):e0248994, 2021.

[16] Kornilia Nikaki, Akinari Sawada, Ahsen Ustaoglu, and Daniel Sifrim. Neuronal control of esophageal peristalsis and its role in esophageal disease. *Current gastroenterology reports*, 21:1–9, 2019.

[17] William G. Paterson, Satish Rattan, and Raj K. Goyal. Esophageal responses to transient and sustained esophageal distension. *American Journal of Physiology-Gastrointestinal and Liver Physiology*, 255(5): G587–G595, 1988.

[18] William G. Paterson. Esophageal peristalsis. *GI Motility Online*, 2006.

[19] Taher I. Omari, Ali Zifan, Charles Cock, and Ravinder K. Mittal. Distension contraction plots of pharyngeal/esophageal peristalsis: next frontier in the assessment of esophageal motor function. *American Journal of Physiology-Gastrointestinal and Liver Physiology*, 323(3):G145–G156, 2022.

[20] Daniel Sifrim and Jafar Jafari. Deglutitive inhibition, latency between swallow and esophageal contractions and primary esophageal motor disorders. *Journal of Neurogastroenterology and Motility*, 18(1):6, 2012.

[21] Julijana Gjorgjieva, Jimena Berni, Jan Felix Evers, and Stephen J. Eglen. Neural circuits for peristaltic wave propagation in crawling drosophila larvae: analysis and modeling. *Frontiers in Computational Neuroscience*, 7:24, 2013.

[22] Cengiz Pehlevan, Paolo Paoletti, and L. Mahadevan. Integrative neuromechanics of crawling in d. melanogaster larvae. *Elife*, 5:e11031, 2016. doi: 10.7554/eLife.11031.

[23] Hugh R. Wilson and Jack D. Cowan. Excitatory and inhibitory interactions in localized populations of model neurons. *Biophysical Journal*, 12(1):1–24, 1972. doi: 10.1016/S0006-3495(72)86068-5.

[24] Peng Du, Niranchan Paskaranandavadivel, Timothy R Angeli, Leo K Cheng, and Gregory O'Grady. The virtual intestine: in silico modeling of small intestinal electrophysiology and motility and the applications. *Wiley Interdisciplinary Reviews: Systems Biology and Medicine*, 8(1):69–85, 2016.

[25] Martyn P. Nash and Alexander V. Panfilov. Electromechanical model of excitable tissue to study reentrant cardiac arrhythmias. *Progress in Biophysics and Molecular Biology*, 85(2-3):501–522, 2004.

[26] Nancy Kopell and G. Bard Ermentrout. Coupled oscillators and the design of central pattern generators. *Mathematical Biosciences*, 90(1-2):87–109, 1988.

[27] Jordan D Chambers, Joel C Bornstein, and Evan A Thomas. Multiple neural oscillators and muscle feedback are required for the intestinal fed state motor program. *PloS one*, 6(5):e19597, 2011.

[28] Peng Du, Greg O'Grady, John B. Davidson, Leo K. Cheng, and Andrew J. Pullan. Multiscale modeling of gastrointestinal electrophysiology and experimental validation. *Critical Reviews in Biomedical Engineering*, 38(3), 2010.
