# Supplementary material for: Modeling based insights into mechanical dysfunction in esophageal motility disorders
Source: PLoS Comput Biol. 2025 Dec 26;21(12):e1013778. doi: 10.1371/journal.pcbi.1013778 (PMC12779157; doi:10.1371/journal.pcbi.1013778)
Supplement: S3 Text — Table A. List of parameters and their values used to obtain the results in Figs 4A and 4B of the main manuscript. Table B. List of parameters and their values used to obtain the results in Fig 4C of the main manuscript. Table C. List of parameters and their values used to obtain the results in Fig 4D of the main manuscript. Table D. List of parameters and their values used to obtain the results in Fig 4E of the main manuscript. Table E. List of parameters and their values used to obtain the results in Fig 4F of the main manuscript. Table F. List of parameters and their values used to obtain the results in Fig 5A of the main manuscript. Table G. List of parameters and their values used to obtain the results in Fig 5B of the main manuscript. Table H. List of parameters and their values used to obtain the results in Fig 6A of the main manuscript. Table I. List of parameters and their values used to obtain the results in Fig 6B of the main manuscript. Table J. List of parameters and their values used to obtain sustained panesophageal contraction. Table K. List of parameters and their values used to obtain the results in Fig 8 of the main manuscript. (PDF) [file pcbi.1013778.s003.pdf]

S3 Text. Parametric values for simulations in the main manuscript

The following section presents tables which list the values of all parameters used for the simulations displayed in the main manuscript.The values of the circuit's parameters (parameters $a - f$) were chosen using a physiologically guided calibration approach. They were chosen to reflect physiologically observed behaviors rather than being derived directly from experimental measurements of synaptic strength, which are currently unavailable for the esophageal neural network. As discussed in the prior supplementary section, unlike the heart, which exhibits intrinsic rhythmicity, the esophagus produces different contraction patterns depending on the type and duration of stimulus. In our model, the synaptic strengths were selected to reproduce key physiological behaviors:

1. **No input:** The system remains at rest, consistent with observations that the esophagus is quiescent in the absence of stimulation.
2. **Short transient input:** Produces a single contraction that propagates along the esophagus, as seen experimentally with brief distension or vagal stimulation.
3. **Sustained input:** Leads to repetitive contractions in the distended region, while preventing propagation beyond the region due to descending inhibition.

Mechanistically, the parameters are set such that sufficient input from mechanoreceptors generates a stable limit cycle, whereas in the absence of input, no oscillations occurs.

Table A: List of parameters and their values used to obtain the results in Fig 4A and Fig 4B of the main manuscript

Parameter	Value	Parameter	Value
ψ	3000	β	100
θ_o	0.05	S_{IC}	2
$\hat{\alpha}$	1.5	x_s	0.1
$\hat{\tau}_\theta$	0.2	$\hat{\tau}_I$	4
a	16	b	20
c	12	d	40
e	15	f	3
w_E	0	w_I	0
ϕ_E	4	ϕ_I	3.7
λ_E	1.3	λ_I	2
g_S	1000	g_E	1000
g_θ	5	$\hat{E}$	0.3
N	70		

The results presented in Figs. 4F, 5A, and 5B are obtained by simulations which solve the neural circuit equations (Eqs. (13), (14), and (15) in the main manuscript). The excitatory inputs from the mechanoreceptors to the excitatory and inhibitory neural populations are not derived from the coupled neuro-mechanical system. Instead, these inputs are assigned predetermined constant values. In these simulations, the neural circuit and its parameters are identical to those used in the baseline solution, which represents a typical esophageal response to FLIP (see Fig 3 in the main manuscript). The only modification is that the location and duration of the distension along the esophagus are adjusted to replicate a short bag and transient distension tests, simulating abrupt deflation.

As such, the tables specifying the parametric values used for these simulations do not include mechanical parameters. Instead, new length and time parameters are introduced. The excitatory input from stretch to the excitatroy neural population is defined as

$$S_E(\chi,\tau) = \begin{cases} w_E & \text{if } \chi_{\text{proximal}} \leq \chi \leq \chi_{\text{proximal}} + l \text{ and } \tau \leq \tau_{\text{deflation}} \\ 0 & \text{otherwise} \end{cases} \tag{1}$$

where χ_{proximal} marks the location of the proximal end of the bag (in case of a regular FLIP simulation, $\chi_{\text{proximal}} = 0$), l denotes the length of the bag ($l = 1$ in a regular FLIP simulation), and $\tau_{\text{deflation}}$ indicates the instant of bag deflation.

Table B: List of parameters and their values used to obtain the results in Fig 4C of the main manuscript

Parameter	Value	Parameter	Value
ψ	3000	β	100
θ_o	0.05	S_{IC}	2
$\hat{\alpha}$	1.5	x_s	0.1
$\hat{\tau}_\theta$	0.2	$\hat{\tau}_I$	4
a	16	b	20
c	12	d	10
e	15	f	3
w_E	1.6	w_I	1.35
ϕ_E	4	ϕ_I	3.7
λ_E	1.3	λ_I	2
g_S	1000	g_E	1000
g_θ	5	$\hat{E}$	0.3
N	70		

Table C: List of parameters and their values used to obtain the results in Fig 4D of the main manuscript

Parameter	Value	Parameter	Value
ψ	3000	β	100
θ_o	0.05	S_{IC}	2
$\hat{\alpha}$	1.5	x_s	0.1
$\hat{\tau}_\theta$	0.2	$\hat{\tau}_I$	4
a	16	b	20
c	12	d	40
e	15	f	3
w_E	1.6	w_I	1.35
ϕ_E	1.6	ϕ_I	3.7
λ_E	1.3	λ_I	2
g_S	1000	g_E	1000
g_θ	5	$\hat{E}$	0.3
N	70		

The excitatory input from stretch to the inhibitory neural population is defined as

$$S_I(\chi,\tau) = \begin{cases} w_I & \text{if } \chi \geq \chi_{\text{proximal}} \text{ and } \tau \leq \tau_{\text{deflation}} \\ 0 & \text{otherwise.} \end{cases} \tag{2}$$

For the simulation shown in Fig 4F, the effect of descending inhibition was removed, resulting in more localized excitation of inhibitory neurons from stretch receptors ($\beta_I = \beta_E$). Consequently, $S_I = 0$ below the distended section of the esophagus, and Eq. (2) becomes

$$S_I(\chi,\tau) = \begin{cases} w_I & \text{if } \chi_{\text{proximal}} \leq \chi \leq \chi_{\text{proximal}} + l \text{ and } \tau \leq \tau_{\text{deflation}} \\ 0 & \text{otherwise.} \end{cases} \tag{3}$$

Table D: List of parameters and their values used to obtain the results in Fig 4E of the main manuscript

Parameter	Value	Parameter	Value
ψ	3000	β	100
θ_o	0.05	S_{IC}	2
$\hat{\alpha}$	1.5	x_s	0.1
$\hat{\tau}_\theta$	0.2	$\hat{\tau}_I$	4
a	16	b	0
c	12	d	0
e	15	f	3
w_E	1.6	w_I	1.35
ϕ_E	4	ϕ_I	3.7
λ_E	1.3	λ_I	2
g_S	1000	g_E	1000
g_θ	5	$\hat{E}$	0.3
N	70		

Table E: List of parameters and their values used to obtain the results in Fig 4F of the main manuscript

Parameter	Value	Parameter	Value
θ_o	0.05	N	70
$\hat{\tau}_\theta$	0.2	$\hat{\tau}_I$	4
a	16	b	20
c	12	d	40
e	15	f	3
w_E	1.6[1]	w_I	1.35[2]
ϕ_E	4	ϕ_I	3.7
λ_E	1.3	λ_I	2
g_S	1000	g_E	1000
g_θ	5	$\hat{E}$	0.3
χ_{proximal}	0.2	l	0.2

1. Excitatory input from mechanoreceptors to the excitatory neural population is defined by Eq. (1). Since there is no deflation, the inequality concerning $\tau_{\text{deflation}}$ is not included.
2. Excitatory input from mechanoreceptors to the inhibitory neural population is defined by Eq. (3). Since there is no deflation, the inequality concerning $\tau_{\text{deflation}}$ is not included.

Table F: List of parameters and their values used to obtain the results in Fig 5A of the main manuscript

Parameter	Value	Parameter	Value
θ_o	0.05	N	70
$\hat{\tau}_\theta$	0.2	$\hat{\tau}_I$	4
a	16	b	20
c	12	d	40
e	15	f	3
w_E	1.6[1]	w_I	1.35[2]
ϕ_E	4	ϕ_I	3.7
λ_E	1.3	λ_I	2
g_S	1000	g_E	1000
g_θ	5	$\hat{E}$	0.3
χ_{proximal}	0.2	l	0.2
$\tau_{\text{deflation}}$	87		

1. Excitatory input from mechanoreceptors to the excitatory neural population is defined by Eq. (1).
2. Excitatory input from mechanoreceptors to the inhibitory neural population is defined by Eq. (2).

Table G: List of parameters and their values used to obtain the results in Fig 5B of the main manuscript

Parameter	Value	Parameter	Value
θ_o	0.05	N	70
$\hat{\tau}_\theta$	0.2	$\hat{\tau}_I$	4
a	16	b	20
c	12	d	40
e	15	f	3
w_E	1.6^1	w_I	1.35^2
ϕ_E	4	ϕ_I	3.7
λ_E	1.3	λ_I	2
g_S	1000	g_E	1000
g_θ	5	$\hat{E}$	0.3
χ_{proximal}	0.2	l	0.2

1. Excitatory input from mechanoreceptors to the excitatory neural population is defined by Eq. (1). Since there is no deflation, the inequality concerning $\tau_{\text{deflation}}$ is not included.
2. Excitatory input from mechanoreceptors to the inhibitory neural population is defined by Eq. (2). Since there is no deflation, the inequality concerning $\tau_{\text{deflation}}$ is not included.

Table H: List of parameters and their values used to obtain the results in Fig 6A of the main manuscript

Parameter	Value	Parameter	Value
ψ	3000	β	100
θ_o	0.05	S_{IC}	2
$\hat{\alpha}$	1.5	x_s	0.1
$\hat{\tau}_\theta$	0.2	$\hat{\tau}_I$	4
a	16	b	20
c	8	d	40
e	12 with $\sigma = 10^1$	f	3
w_E	1.6	w_I	1.35
ϕ_E	4	ϕ_I	3.7
λ_E	1.3	λ_I	2
g_S	1000	g_E	1000
g_θ	5	$\hat{E}$	0.3
N	70		

1. Irregularities introduced to parameter e with mean = 12 and standard deviation $\sigma = 10$.

Table I: List of parameters and their values used to obtain the results in Fig 6B of the main manuscript

Parameter	Value	Parameter	Value
ψ	3000	β	100
θ_o	0.05	S_{IC}	2
$\hat{\alpha}$	1.5	x_s	0.1
$\hat{\tau}_\theta$	0.2	$\hat{\tau}_I$	4
a	16	b	20
c	8	d	40
e	12	f	3
w_E	1.6 with $\sigma = 0.75^1$	w_I	1.35
ϕ_E	4	ϕ_I	3.7
λ_E	1.3	λ_I	2
g_S	1000	g_E	1000
g_θ	5	$\hat{E}$	0.3
N	70		

1. Irregularities introduced to parameter w_E with mean = 1.6 and standard deviation $\sigma = 0.75$.

Table J: List of parameters and their values used to obtain sustained panesophageal contraction

Parameter	Value	Parameter	Value
ψ	3000	β	100
θ_o	0.05	S_{IC}	2
$\hat{\alpha}$	1.5	x_s	0.1
$\hat{\tau}_\theta$	0.2	$\hat{\tau}_I$	4
a	16	b	20
c	12	d	40
e	0	f	3
w_E	1.6	w_I	1.35
ϕ_E	4	ϕ_I	3.7
λ_E	1.3	λ_I	2
g_S	1000	g_E	1000
g_θ	5	$\hat{E}$	0.3
N	70		

Table K: List of parameters and their values used to obtain the results in Fig 8 of the main manuscript

Parameter	Value	Parameter	Value
ψ	3000	β	100
θ_o	0.05	S_{IC}	2
$\hat{\alpha}$	1.5	x_s	0.1
$\hat{\tau}_\theta$	0.2	$\hat{\tau}_I$	4
a	16	b	20
c	12	d	40
e	15	f	3
w_E	1.3	w_I	0
ϕ_E	4	ϕ_I	3.7
λ_E	1.3	λ_I	2
g_S	1000	g_E	1000
g_θ	5	$\hat{E}$	0.3
N	70		
