# Supplementary material for: Modeling based insights into mechanical dysfunction in esophageal motility disorders
Source: PLoS Comput Biol. 2025 Dec 26;21(12):e1013778. doi: 10.1371/journal.pcbi.1013778 (PMC12779157; doi:10.1371/journal.pcbi.1013778)
Supplement: S4 Text — Fig A. Solution of sensitivity test to varying the value of parameter e. (a) Color-coded topography of muscle contraction pattern (θ) with the parameter e modified to 0.7e, representing a 30% decrease from the original value of e. (b) Color-coded topography of muscle contraction pattern (θ) with the parameter e modified to 1.2e, representing a 20% increase from the original value of e. (c) Plot of the intersegmental phase lag as a function of percentage deviation from the baseline value of parameter e where 0% corresponds to the baseline e value (e = 15). Fig B. Solution of sensitivity test to varying the value of parameter f. (a) Color-coded topography of muscle contraction pattern (θ) with the parameter f modified to 0.5f, representing a 50% decrease from the original value of f. (b) Color-coded topography of muscle contraction pattern (θ) with the parameter f modified to 2f, representing a 100% increase from the original value of f. (c) Plot of the intersegmental phase lag as a function of percentage deviation from the baseline value of parameter f where 0% corresponds to the baseline f value (f = 3). Fig C. Solution of sensitivity test to varying the value of parameter wI. (a) Color-coded topography of muscle contraction pattern (θ) with the parameter wI modified to 0.5wI, representing a 50% decrease from the original value of wI. (b) Color-coded topography of muscle contraction pattern (θ) with the parameter wI modified to 1.2wI, representing a 20% increase from the original value of wI. (c) Plot of the contraction propagating speed as a function of percentage deviation from the baseline value of parameter wI where 0% corresponds to the baseline wI value (wI = 1.35). Fig D. Solution of sensitivity test to varying the value of parameter b. (a) Plot of the intersegmental phase lag as a function of percentage deviation from the baseline value of parameter b where 0% corresponds to the baseline b value (b = 20). (b) Plot of the segment activity duration (how long a [file pcbi.1013778.s004.pdf]

S4 Text. Sensitivity testing and robustness analysis

In the pursuit of a comprehensive understanding of the dynamics within the coupled model, we perform a parametric study. This study involves systematically varying individual parameters by increments of ±5, ±10, ±15, ±20%, allowing us to dissect the relationship between these parameters and the model's response. For some parameters, greater increments are considered. The parametric study or sensitivity test has a dual purpose. Firstly, to evaluate the robustness of the model to varying parameters. Secondly, to explore how different parameters affect the solution to the extent that the emerging response resembles an esophageal motility disorder. The impact of varying individual parameters on the simulation results is assessed both qualitatively and quantitatively, providing a comprehensive view of the model's behavior under various conditions.

The quantitative evaluation focuses on the following criteria:

- Maximum values of E and I – amplitude of E and I oscillations.
- Period of E and I oscillations.
- Contraction duration (length/wave speed) – how long it takes the wave to propagate along the entire length.
- Intersegmental phase lag – time delay in the propagation of the neural signal between adjacent segments.
- Segment activity duration – how long an oscillator is active.

The qualitative assessment is concerned with examining the color-coded topography of the solution, and comparing it with the one of the baseline case (Fig 3 in main manuscript). Are there repetitive, forward propagating contractions? If so, do they decay with time or remain intact as long as distension is applied?

The results of the sensitivity testing reveal that the model is in fact robust to varying individual parameters. Additionally, it shows that the model is more sensitive to some parameters over others. Lastly, the results uncover interesting patterns which provide insights into the role each parameter plays in creating the desired RACs pattern, with the constrains discussed in the main manuscript. Samples for the qualitative and quantitative analysis are presented graphically or through topographic maps in Figs A-E.

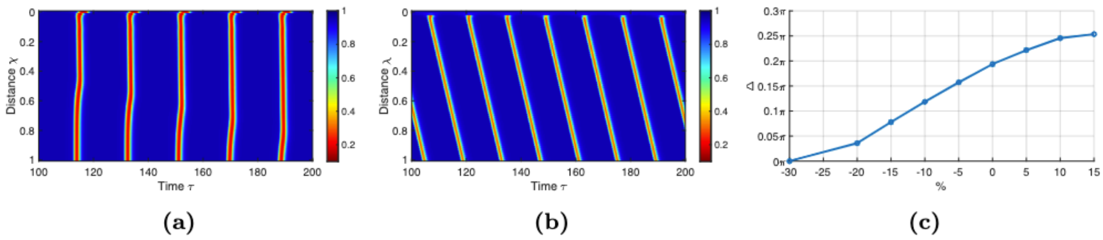

Fig A. **Solution of sensitivity test to varying the value of parameter e. (a)** Color-coded topography of muscle contraction pattern (θ) with the parameter e modified to $0.7e$, representing a 30% decrease from the original value of e. **(b)** Color-coded topography of muscle contraction pattern (θ) with the parameter e modified to $1.2e$, representing a 20% increase from the original value of e. **(c)** Plot of the intersegmental phase lag as a function of percentage deviation from the baseline value of parameter e where 0% corresponds to the baseline e value ($e = 15$).

Fig A displays the simulations solutions to different values of parameter e. Reducing the value of parameter e implies less inhibition of excitatory neurons. Thus, increasing overall excitatory activity. Reducing the value of parameter e is translated into uniform or fast propagating contractions, and decrease intersegmental phase lag (Fig Aa). We explain this observation through the following concepts. When excitation is increased, excitatory neurons fire more frequently and with greater intensity, leading to a stronger signal being transmitted along the neural pathways. Stronger signal can overcome the differences in physical properties between segments, and propagate more quickly along the neural pathways, thus, reduce intersegmental phase lag. For the exact same reason, we see an opposite trend for increasing the value of parameter e (reducing excitatory activity). As Fig Ab indicates, increasing the value of parameter e results in greater phase lag.

Fig B presents the results obtained by the simulations with different values of parameter f. Since parameter f is responsible for the inhibition of inhibitory neuronal population, the trends observed by

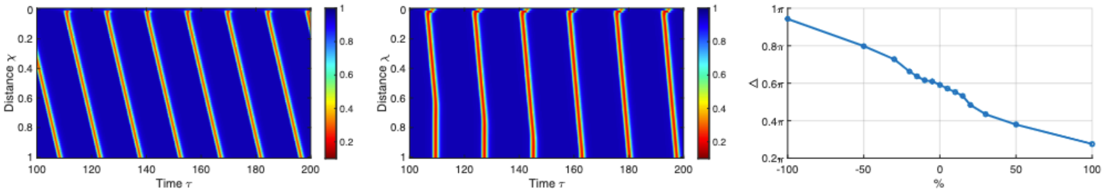

(a) **(b)** **(c)**

Fig B. **Solution of sensitivity test to varying the value of parameter f. (a)** Color-coded topography of muscle contraction pattern (θ) with the parameter f modified to $0.5f$, representing a 50% decrease from the original value of f. **(b)** Color-coded topography of muscle contraction pattern (θ) with the parameter f modified to $2f$, representing a 100% increase from the original value of f. **(c)** Plot of the intersegmental phase lag as a function of percentage deviation from the baseline value of parameter f where 0% corresponds to the baseline f value ($f = 3$).

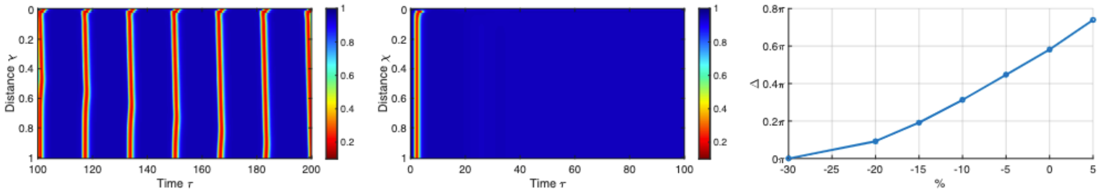

(a) **(b)** **(c)**

Fig C. **Solution of sensitivity test to varying the value of parameter w_I. (a)** Color-coded topography of muscle contraction pattern (θ) with the parameter w_I modified to $0.5w_I$, representing a 50% decrease from the original value of w_I. **(b)** Color-coded topography of muscle contraction pattern (θ) with the parameter w_I modified to $1.2w_I$, representing a 20% increase from the original value of w_I. **(c)** Plot of the contraction propagating speed as a function of percentage deviation from the baseline value of parameter w_I where 0% corresponds to the baseline w_I value ($w_I = 1.35$).

varying its value are opposite to the ones observed by varying the value of parameter e. As shown in Fig B, increasing the value of parameter f results in fast contractions whereas decreasing the value of parameter f results in slower propagation (more slanted contractions). Increasing the value of parameter f implies less excitation of inhibitory neurons, thus less inhibition of excitatory population, and therefore a more uniform firing.

Fig C displays the results of simulations obtained by using various values of parameter w_I. These results demonstrate that reducing this parameter, indicating less overall inhibition, leads to a significant decrease in intersegmental phase lag. This emerging observation is in line with our expectations. Increasing the value of parameter w_I results in a single contraction which quickly decays. Thus, increasing the value of parameter w_I results in an absent contractile response. Initially, there is a spike, known as the excitable regime, which quickly decays, and the system converges into its resting state. This outcome is due to the fact that the w_I value selected for the baseline case ($w_I = 1.35$) is close to the upper limit for this parameter for which a limit cycle solution exists. Increasing the value of parameter w_I shifts the I nullcline (represented by the black curve in Fig B in S1 Text) to the left on the phase diagram.

Other interesting parameters are b and d, which were discussed in great details in [1] and [2]. Figures D and E present the results obtained by simulations with different b and d values, respectively. Fig Da shows that increasing the value of parameter b is translated into reduction of intersegmental phase lag. This is expected given that increasing b implies greater excitation. However, varying b has an additional, much more obvious effect on the solution, shown in Fig Db. Increasing b results in a stronger and longer contraction, as segment activity duration increases. Reducing the value of parameter b leads to an opposite pattern. Lower b values reduces excitatory signal, leading very weak contractions. These patterns can be observed qualitatively in Fig Dc and Fig Dd.

An opposite trend is detected when increasing or decreasing the value of parameter d. Increasing the value of parameter d is equivalent to increasing inhibitory signal to excitatory population from nearest-neighbor. Thus, resulting in a weaker and shorter contraction duration, as segment activity duration decreases. This is presented qualitatively in Fig E.

Both [1] and [2] concluded that increasing intersegmental coupling (values of parameters b and d) has a stabilizing effect on propagation, where larger values of b and d increase the model's robustness. However,

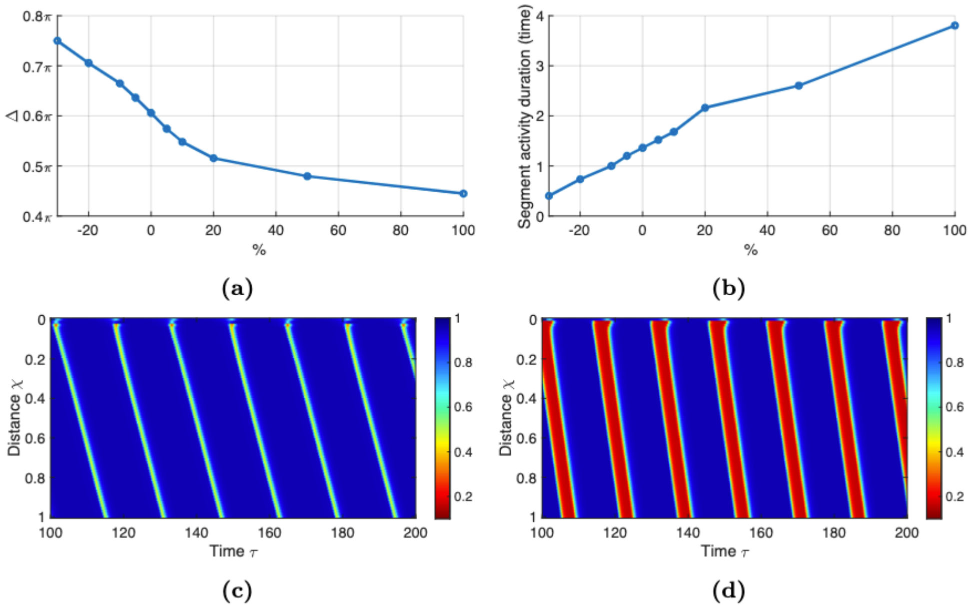

Fig D. **Solution of sensitivity test to varying the value of parameter b.** **(a)** Plot of the intersegmental phase lag as a function of percentage deviation from the baseline value of parameter b where 0% corresponds to the baseline b value ($b = 20$). **(b)** Plot of the segment activity duration (how long an oscillator is active) as a function of percentage deviation from the baseline value of parameter b. Due to the relation between muscle contraction pattern (θ) and the excitatory activity level (E) displayed in Eq. (8) in the main manuscript, segment activity duration is directly related to contraction strength. **(c)** Color-coded topography of muscle contraction pattern (θ) with the parameter b modified to $0.7b$, representing a 30% decrease from the original value of b. **(d)** Color-coded topography of muscle contraction pattern (θ) with the parameter b modified to $2b$, representing a 100% increase from the original value of b.

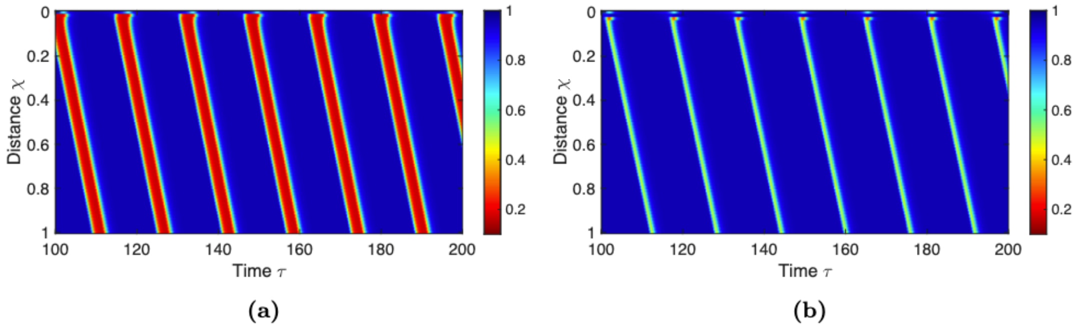

Fig E. **Solution of sensitivity test to varying the value of parameter d.** **(a)** Color-coded topography of muscle contraction pattern (θ) with the parameter d modified to $0.5d$, representing a 50% decrease from the original value of d. **(b)** Color-coded topography of muscle contraction pattern (θ) with the parameter d modified to $1.5d$, representing a 50% increase from the original value of d.

our scenario has additional constrains that were not considered in previous work, which requires to set an upper limit to the possible values of b and d . Increasing b and d reduces the impact or weighted contribution of the other parameters (such as w_I and w_E) on the emerging solution. The interplay between b and w_I for example is essential for capturing the range of esophageal response to distension, discussed in the main manuscript. Increasing the value of b beyond a certain threshold can cause the signal to propagate even without the presence of distension (a solution which looks like Fig 4(F) rather than Fig 5(B)).

Lastly, the system's response to the change of strength of input parameter to excitatory population, w_E, has also been examined. The results of this investigation are at the center of a different study, which we encourage the readers to visit [3].

As indicated in the main text, biological systems are naturally prone to variation, making it essential to test the model's ability to withstand minor disruptions without altering its contraction pattern. To achieve this, we systematically assess the model's robustness by introducing small irregularities into its parameters

along the length. For each parameter, we randomly sample values from a Gaussian distribution with specific mean and standard deviation, and examine whether the model can maintain its contraction pattern when subjected to these parameter variations. Several examples are presented in Fig F. The figure indicates that the model is robust for the parametric values chosen as the baseline solution. Note that variability may not come from randomness but due to structural changes in the esophagus.

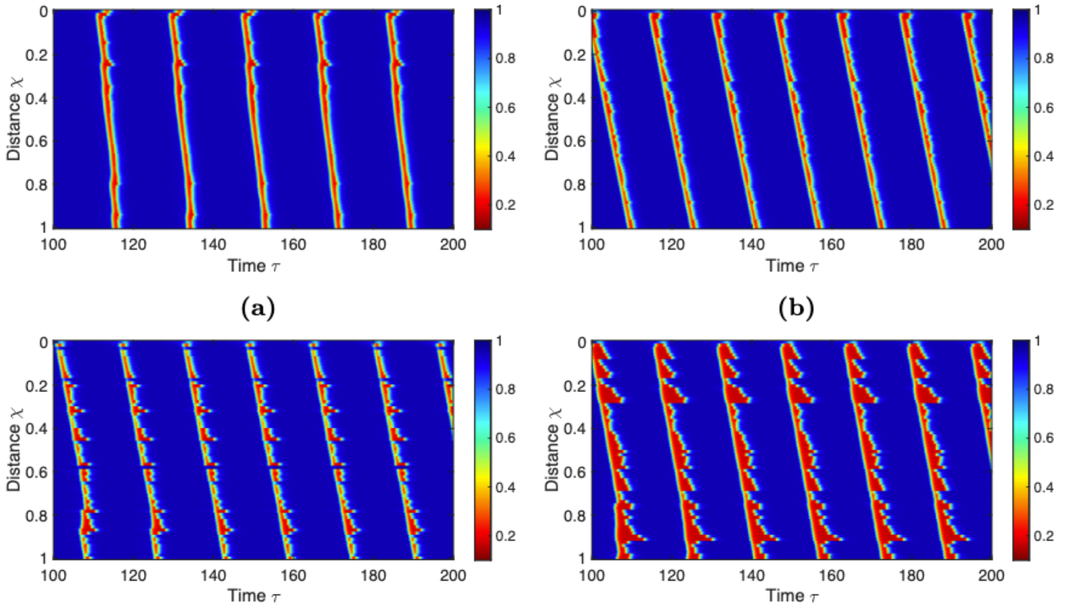

Fig F. **Color-coded muscle contraction pattern (θ) topography obtained by the neuromechanical model with randomly sample values from a Gaussian distribution with specific mean and standard deviation.** **(a)** Irregularities introduced to c with mean=12 and standard deviation = 3. **(b)** Irregularities introduced to e with mean=15 and standard deviation = 10. **(b)** Irregularities introduced to d with mean=40 and standard deviation = 20. **(d)** Irregularities introduced to b with mean=20 and standard deviation = 10.

References

[1] Julijana Gjorgjieva, Jimena Berni, Jan Felix Evers, and Stephen J. Eglen. Neural circuits for peristaltic wave propagation in crawling drosophila larvae: analysis and modeling. *Frontiers in Computational Neuroscience*, 7:24, 2013.

[2] Cengiz Pehlevan, Paolo Paoletti, and L. Mahadevan. Integrative neuromechanics of crawling in d. melanogaster larvae. *Elife*, 5:e11031, 2016. doi: 10.7554/eLife.11031.

[3] Guy Elisha, Richard Gast, Sourav Halder, Sara A. Solla, Peter J. Kahrilas, John E. Pandolfino, and Neelesh A. Patankar. Direct and retrograde wave propagation in unidirectionally coupled wilson-cowan oscillators. *Phys. Rev. Lett.*, 134:058401, Feb 2025. doi: 10.1103/PhysRevLett.134.058401. URL `https://link.aps.org/doi/10.1103/PhysRevLett.134.058401`.
