# Supplementary material for: Modeling based insights into mechanical dysfunction in esophageal motility disorders
Source: PLoS Comput Biol. 2025 Dec 26;21(12):e1013778. doi: 10.1371/journal.pcbi.1013778 (PMC12779157; doi:10.1371/journal.pcbi.1013778)
Supplement: S5 Text — (PDF) [file pcbi.1013778.s005.pdf]

S5 Text. Numerical origin of the apparent discontinuity in simulated CSA

In Fig 4B of the main manuscript, the simulated cross-sectional area (CSA) value appears to drop instantaneously from dark blue (CSA=2.5) to light blue (CSA=2.0). The sudden drop in CSA value arises as a result of the simulation time step. It can be explained through the combined effects of the simulation's esophageal wall stiffness and the viscosity of the fluid within the FLIP device. The following discussion, which explains this observation, has been studied in great detail in [1, 2].

During a traveling contraction wave, the extent and spatial distribution of this expansion depend on the relative contributions of wall stiffness and fluid viscosity. When the tube is compliant and the fluid highly viscous, resistance to flow is substantial, resulting in asymmetric expansion—greater downstream than upstream of the contraction. Conversely, when the tube is stiff and the fluid viscosity is low, as in the FLIP device and esophageal simulations, expansion remains nearly uniform along the length of the tube. In this regime, instead of the contraction pushing fluid forward, the fluid flows through the contraction, since more force is needed to expand the tube than resisting this flow. As the contraction exits the computational domain, the displaced fluid redistributes, restoring the CSA to its baseline value throughout the tube. Because the simulated fluid viscosity is low, this recovery occurs nearly instantaneously. However, the numerical time step used in the simulation is not sufficiently small to capture this rapid transient, and therefore the brief adjustment in fluid volume and pressure appears as a discontinuity or darker streak in the CSA field.

References

[1] Shashank Acharya, Wenjun Kou, Sourav Halder, Dustin A. Carlson, Peter J. Kahrilas, John E. Pandolfino, and Neelesh A. Patankar. Pumping patterns and work done during peristalsis in finite-length elastic tubes. *Journal of Biomechanical Engineering*, 143(7), Mar 2021. ISSN 1528-8951. doi: 10.1115/1.4050284.

[2] Guy Elisha, Shashank Acharya, Sourav Halder, Dustin A. Carlson, Wenjun Kou, Peter J. Kahrilas, John E. Pandolfino, and Neelesh A. Patankar. Peristaltic regimes in esophageal transport. *Biomechanics and Modeling in Mechanobiology*, pages 1–19, November 2022. doi: 10.1007/s10237-022-01625-x.
